# M102 activates both NRF2 and HSF1 transcription factor pathways and is neuroprotective in cell and animal models of amyotrophic lateral sclerosis

**DOI:** 10.1186/s13024-025-00908-y

**Published:** 2025-11-04

**Authors:** Amy F. Keerie, Raquel Rua Martins, Chloe F. Allen, Katie Bowden, Sufana Al Mashhadi, Thomas Marlow, Monika Myszczynska, Nikitha Thakur, Selina N. Beal, Allan Shaw, Shivani Suresh, Scott N. McKinnon, Johnathan Cooper-Knock, Ryan J. H. West, Sam Bonsall, Alex Daniel, Tyler Wells, Vedanth Kumar, Brittany C. S. Ellis, Maureen Higgins, Albena T. Dinkova-Kostova, Tatyana A. Shelkovnikova, Ira N. Kalfus, Ning Shan, Pamela J. Shaw, Laura Ferraiuolo, Richard J. Mead

**Affiliations:** 1 Sheffield Institute for Translational Neuroscience, University of Sheffield, 385 Glossop Road, Sheffield, S10 2HQ UK; 2 The Neuroscience Institute, University of Sheffield, 385 Glossop Road, Sheffield, S10 2HQ UK; 3 Aclipse Therapeutics, 411 Swedeland Road, Suite 23-1080, King of Prussia, PA 19406 USA; 4 NIHR Sheffield Biomedical Research Centre, Sheffield Teaching Hospitals NHS Foundation Trust, Glossop Road, Sheffield, S10 2JF UK; 5 Jacqui Wood Cancer Centre, Division of Cancer Research, Ninewells Hospital and Medical School, University of Dundee, Dundee, DD1 9SY UK

**Keywords:** S-apomorphine, M102, NRF-2 activation, HSF1 activation, Neuroprotection, Amyotrophic lateral sclerosis

## Abstract

M102 is a central nervous system (CNS) penetrant small molecule electrophile which activates in vivo the NF-E2 p45-related factor 2-antioxidant response element (NRF2-ARE) pathway, as well as transcription of heat-shock element (HSE) associated genes. In the TDP-43^Q331K^ transgenic mouse model of ALS dosed subcutaneously at 5 mg/kg OD or 2.5 mg/kg BD with M102, significant improvements in compound muscle action potential (CMAP) amplitude of hind limb muscles and gait parameters were observed at 6 months of age, with associated target engagement. An oral dose response study of M102 in *SOD1*^G93A^ transgenic mice showed a dose-dependent improvement in CMAP of hindlimb muscles which correlated with preservation of lumbar spinal motor neurons at the same time point. These data enabled prediction of human efficacious exposures and doses, which were well within the safety margin predicted from Good Laboratory Practice (GLP) toxicology studies. A parallel program of work in vitro showed that M102 rescued motor neuron survival in co-culture with patient-derived astrocytes from sporadic, *C9orf72* and *SOD1* ALS cases. Markers of oxidative stress, as well as indices of TDP-43 proteinopathy were also reduced by exposure to M102 in these in vitro models. This comprehensive package of preclinical efficacy data across two mouse models as well as patient-derived astrocyte toxicity assays, provides a strong rationale for clinical evaluation of M102 in ALS patients. Combined with the development of target engagement biomarkers and the completed preclinical toxicology package, a clear translational pathway to testing in ALS patients has been developed.

**Supplementary Information:**

The online version contains supplementary material available at 10.1186/s13024-025-00908-y.

## Introduction

Amyotrophic lateral sclerosis (ALS) is a rapidly progressive, fatal neurodegenerative disorder in which motor neuron injury and cell death causes muscle weakness and wasting, leading to progressive loss of motor control of the upper and lower limbs as well as bulbar and respiratory functions. The lifetime risk is approximately 1 in 300–350 [1] and the average course of the disease is 2.5-3 years from symptom onset [2]. Currently, there are no effective treatments to halt or reverse the progression of ALS and approved drugs only marginally increase survival (riluzole) or disease progression (edaravone) [3–5]. The anti-sense oligonucleotide (ASO) treatment tofersen (QALSODY) has beneficial clinical effects and biomarker readouts, but applies only to the ~ 2% of ALS patients who harbor a SOD1 mutation [6]. A key feature of ALS is the speed of progression. This poses huge problems of adjustment for affected individuals, an escalating burden on carers and families, and a challenge to those purchasers and providers of healthcare who are involved in meeting the variable, rapidly changing and complex care needs [7, 8].

The pathophysiology of ALS is complex. More than 30 genes are known to cause or contribute to motor neuron degeneration in ALS [9]. Even in the presence of a mutation in a gene such as *SOD1*, the structure and function of which are well understood, it is recognised that a cascade of multiple pathophysiological mechanisms within both motor neurons and neighbouring glial cells contribute to neurodegeneration [9–11]. Data from disease model systems and human biosamples provide strong evidence for a role of redox imbalance, inflammation, mitochondrial dysfunction and altered proteostasis, as key drivers of the pathobiology of ALS [12–16]. Therapies targeting individual pathways have failed in the clinic or have shown marginal efficacy. In addition, few studies in ALS have shown target engagement for the proposed therapeutic agent in the clinic [17] with the exception of tofersen [6]. There is clearly a huge unmet need for effective neuroprotective therapies to slow disease progression in ALS.

Transcription factor NF-E2 p45-related factor 2 (NRF2) is a master regulator of the antioxidant response and activates the expression of over 1000 genes with cytoprotective properties [18–20]. NRF2 protein levels are highly regulated through several different mechanisms including negative regulation at the protein level by Kelch-like ECH-associated protein 1 (KEAP1) [21]. There is a body of evidence from both cellular and animal models and human biosamples that this cytoprotective NRF2 system is dysregulated in ALS [22–27].

We previously identified M102 (chemical name: S-(+)-10,11-dihydroxyaporphine) in a compound screen to discover blood brain barrier penetrant NRF2-ARE pathway activators [28]. We reported that M102 enhanced glutathione (GSH) secretion from astrocytes in co-culture; protected neuromuscular junctions from denervation in *SOD1*^G93A^ mice; and slowed the decline in motor function when dosed at 5 mg/kg subcutaneously daily. M102 also reduced the elevated basal oxidative stress seen in fibroblasts from ALS cases [28]. M102 (S[+]-apomorphine) is an enantiomer of R-apomorphine which is used as a dopamine agonist in Parkinson’s disease. M102 itself is a very weak dopamine antagonist [29] and its structure is consistent with known NRF2 activators predicted to function through modification of cysteine residues on KEAP1, thereby reducing the degradation of NRF2, increasing NRF2 translocation to the nucleus and upregulating the expression of multiple cytoprotective genes [18, 20]. M102, which we now demonstrate activates both the NRF2 and HSF1 transcription factor pathways, has the potential to modulate all four of the key drivers of neurodegeneration highlighted above. By targeting multiple pathways, we expect to significantly increase the probability of neuroprotection of motor neurons and clinical success.

Lack of effective translation between mouse models and clinical trials has hampered ALS treatment discovery over the last 20 years, with many compounds showing potential in mouse models, but failing to show efficacy in human clinical trials [17, 30, 31]. Here, we have generated an encouraging pre-clinical data set demonstrating significant positive effects on the same behavioural outputs in two robust and reproducible ALS mouse models, as well as a preclinical toxicology package that predicts safe efficacious doses in humans. In addition, we provide evidence that M102 increases motor neuron survival in an in vitro model of patient-derived astrocyte toxicity [10] across multiple subtypes of ALS. M102 reduces toxicity from astrocytes derived from *C9orf72*,* SOD1* and sporadic ALS patients and results in neuroprotection of co-cultured motor neurons. Overall, to our knowledge, this is one of the most comprehensive packages of pre-clinical efficacy for therapy development in ALS.

## Results

### M102 activates NRF2 and HSF1 transcription factor pathways in vivo

We previously identified M102 in a screen for CNS penetrant NRF2 activating molecules. We demonstrated that M102 activated NRF2-directed transcription in vitro and in the CNS and was able to rescue motor deficits in the *SOD1*^G93A^ mouse model of ALS at a daily subcutaneous dose of 5 mg/kg [28]. In vitro evidence showed that M102 was able to increase production of glutathione from astrocytes, thereby mediating neuroprotection to co-cultured motor neurons. In order to expand the preclinical validation to other genetic subtypes of ALS we set out to establish a dose response for NRF2 activation in the CNS as a precursor to investigating efficacy in a second, *TARDBP* mutant ALS mouse model for which we have validated readouts [32].

Electrophilic compounds such as M102 also have the potential to activate HSF1 (heat shock factor 1) signalling pathways [33, 34] and since HSF1 activation would address additional pathogenic mechanisms in ALS [35], we also sought to establish whether M102 was able to transcriptionally activate canonical HSF1 targets in vitro and in vivo as well as NRF2-related targets.

We treated HeLa-HSE-Luciferase cells expressing luciferase under the transcriptional control of the HSP70.1 promoter with varying concentrations of M102, or M102 in the presence of CuCl_2_ to mimic oxidation in vivo, and showed that M102 was a robust activator of the HSP70.1 promoter (Suppl. Figure 1 A, B). Western blotting of lysates of SHSY5Y cells, showed that M102 increased expression of both the NRF2 target NAD(P)H quinone dehydrogenase 1 (NQO1) and the HSF1 target HSP70 (Hspa1a), and that siRNA treatment reducing HSF1 or NRF2 was able to ablate the response for their respective targets (Suppl. Figure 1 C, D).

**Fig. 1:**
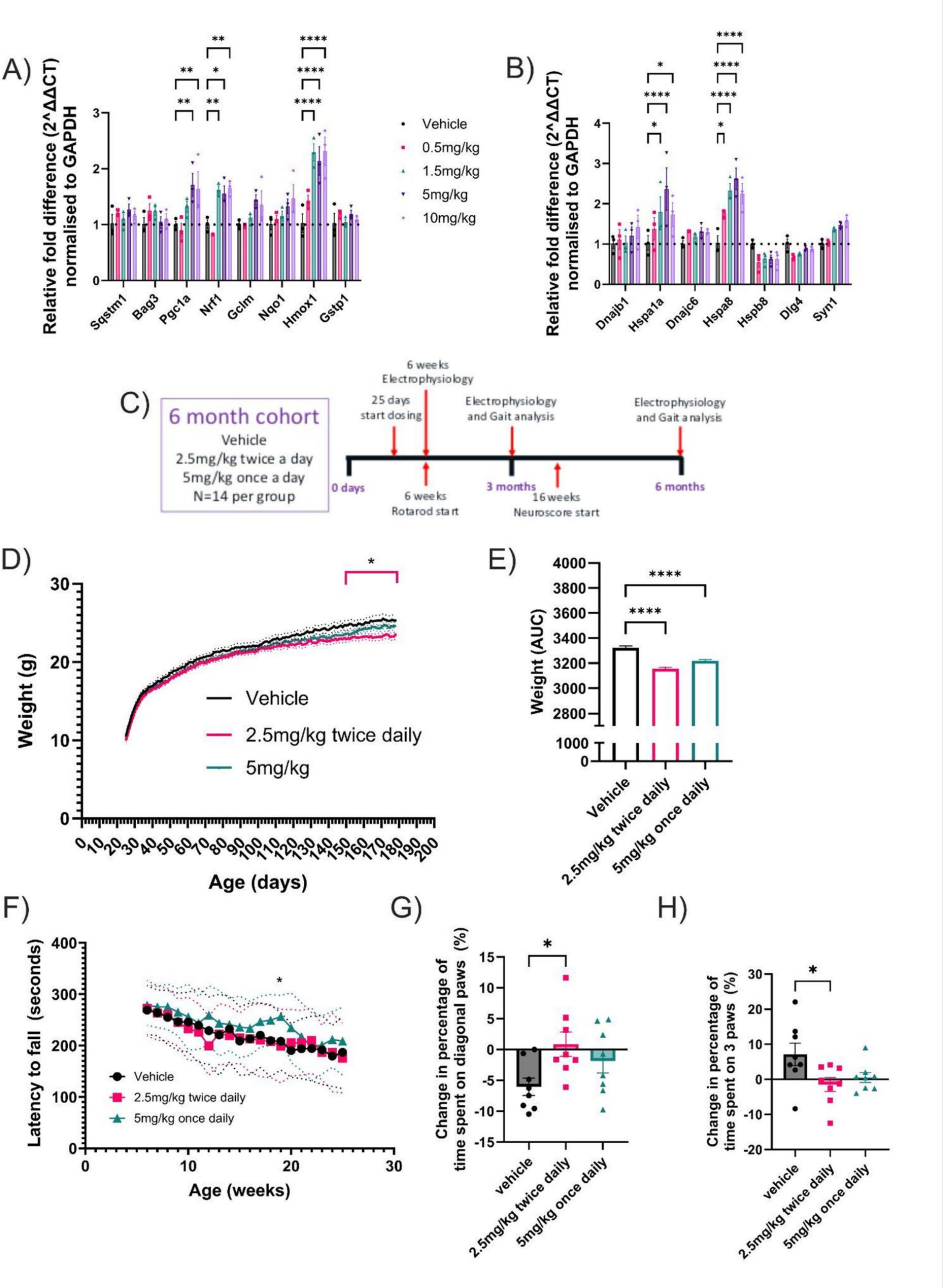
M102 activates NRF2 and HSF1 in WT mice and attenuates disease progression in TDP-43^Q331K^ mice.Changes in: **A**) NRF2 and **B**) HSF1 targets 24 h post dose after 7 days of dosing subcutaneously with different concentrations of M102 (Two-way ANOVA with Dunnett’s multiple comparison test. *N* = 3 per group). **C**) Diagram showing the design of the TDP-43^Q331K^ study with the major timepoints for electrophysiology and gait analysis. **D**) Body weight over time showing a reduction in the 2.5 mg/kg BD M102 group when compared to vehicle dosed (Two-way ANOVA with Dunnett’s multiple comparison test. *N* = 13–14 per group). Dotted lines show +/- SEM. **E**) Area under the curve analysis of body weight showing reduction in both M102 dosed groups when compared to the vehicle dose group (One-way ANOVA with Dunnett’s multiple comparison test. *N* = 13–14). **F**) Rotarod data shown as latency to fall (Two-way ANOVA with Dunnett’s multiple comparison test. Data shown as mean with dotted lines showing +/- SD, *N* = 13–14 per group). Change in time spent on: **G**) Diagonal paws and **H**) 3 paws showing significant improvement of the 2.5 mg/kg BD M102 group when compared to the vehicle group (two-way ANOVA with Dunnett’s multiple comparison test. *N* = 8 per group). All data shown as mean +/- SEM unless specified. Significance: * = *p* < 0.05, ** = *p* < 0.01, *** = *p* < 0.001, **** = *p* < 0.0001

To confirm activation of both pathways in vivo, we dosed wild-type (WT) mice with M102 at 0.5, 1.5, 5 and 10 mg/kg SC, once daily for 7 days, and measured the transcriptional response in cerebral cortex tissue using RT-qPCR. Figure 1 shows the transcriptional response of NRF2 (Fig. 1A) and HSF1 (Fig. 1B) regulated genes. Gene targets of both transcription factors showed a clear dose response, with 5 mg/kg being optimal in most cases. This represents the first indication that M102 is also able to activate HSF1 directed transcription in vivo, with HSF1 regulated genes including Hspa1a (HSP70) and Hspa8 showing elevated transcriptional responses.

### M102 rescues motor, weight and, neurophysiological phenotypes in the TDP-43^Q331K^ Transgenic mouse model of ALS

The study design for evaluating the effect of M102 in hu*TDP-43*^Q331K^ transgenic mice is shown in Fig. 1C and is based on our previous phenotypic characterisation of this ‘low’ TDP-43 expressing model which shows a relatively mild phenotype compared to TDP-43 overexpressing models [32] We established a protocol in this model which utilised female mice only, since they show the same but less variable phenotype compared to male mice [32]. Female *TDP-43*^Q331K^ mice were block randomised into three dosing groups and dosed subcutaneously with 5 mg/kg M102 as a split dose twice daily at 2.5 mg/kg twice daily (BD) or a single 5 mg/kg once daily (OD) dose from 25 days until 6 months of age. This 6 month timepoint was selected based on our previous published study design as later timepoints did not evidence significant progression beyond that seen at 6 months of age [32]. A variety of behavioural tests were performed at time points previously shown to correlate with significant milestones of disease progression, and CNS tissue was collected at 3 and 6 months.

Transgenic *TDP-43*^Q331K^ mice gain significantly more weight compared to their non-transgenic littermates [32]. This is due to the increased amount of food they consume and the reduction in activity seen with this mutation in *TDP-43* that is linked to an apathy fronto-temporal dementia (FTD) phenotype [36]. Mouse weights were recorded daily before dosing to determine dose volume. Figure 1D shows that weight gain is significantly reduced from 161 days in the 2.5 mg/kg BD group (two-way ANOVA with Dunnett’s post-test). At the end of the study there was a significant decrease in weight of the M102 2.5 mg/kg BD dosed mice when compared to vehicle controls. At 177 days of age, M102 2.5 mg/kg BD dosed animals weighed 23.2 +/- 1.9 g compared to vehicle animals that weighed 25.4 +/- 2.3 g (*p* = 0.03). When analysed as area under the curve, weight is significantly reduced in both 5 mg/kg and 2.5 mg/kg BD dosed groups (Fig. 1E; one-way ANOVA with Dunnett’s post-test). Rotarod performance was also improved in the 5 mg/kg dose group at 19 weeks of age (Fig. 1F -two- way ANOVA with Dunnett’s post-testing).

Gait analysis was carried out at 3 and 6 months of age. An improvement in gait (Fig. 1G, H) was observed in the 2.5 mg/kg BD dosed group when compared to vehicle controls, using Catwalk (NoldusXT) gait analysis. This was shown as an increase in the amount of time spent using diagonal paws, a marker of normal mouse gait, versus a decrease in the amount of time spent on three paws, suggesting a reduction of gait unsteadiness (one-way ANOVA, with Dunnett’s post-test). The 5 mg/kg OD group showed a similar numerical improvement in gait although this did not reach statistical significance.

**Fig. 2:**
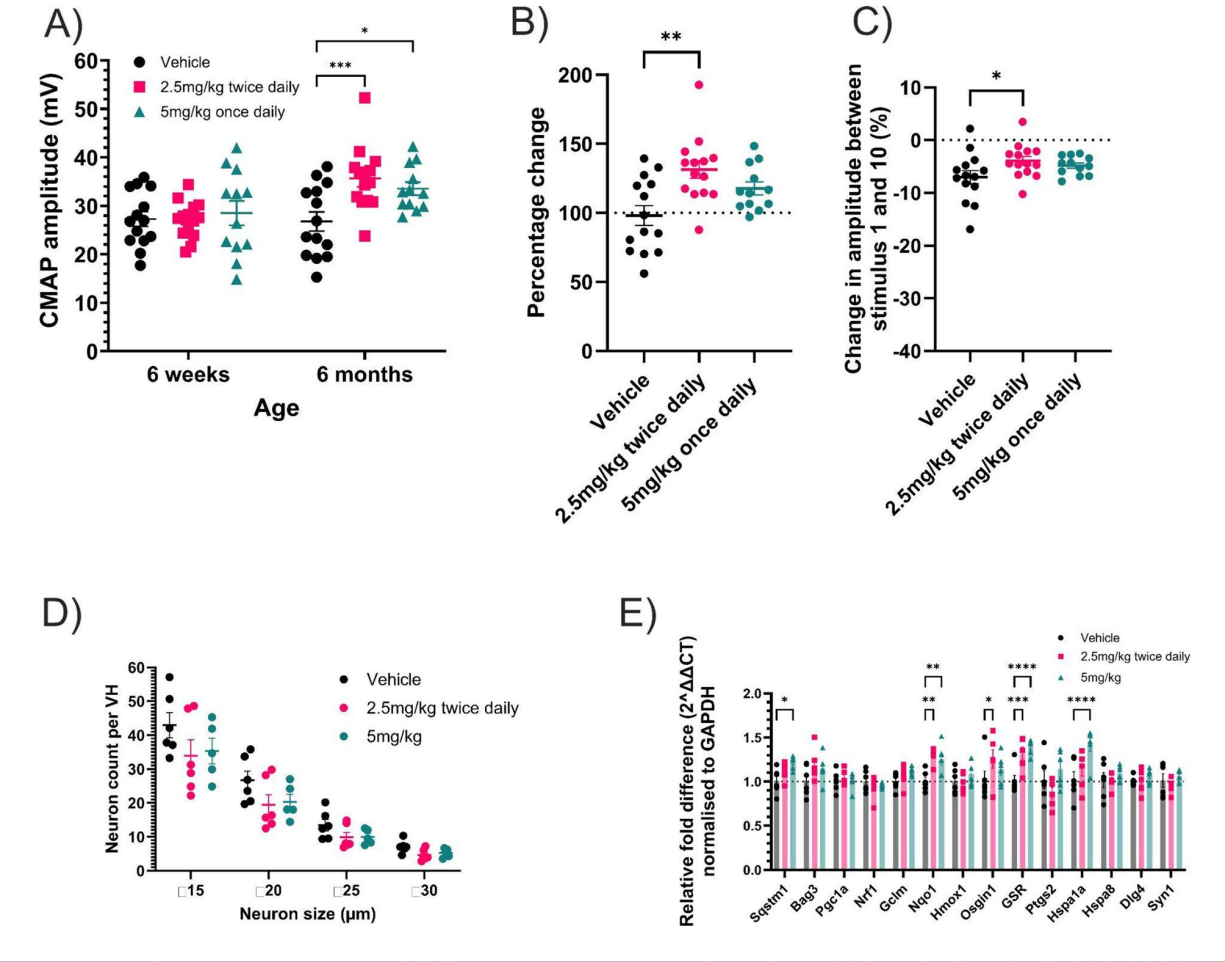
M102 treatment improves electrophysiological parameters in TDP-43^Q331K^ mice. **A**) Compound muscle action potential (CMAP) amplitude of the hindlimb muscle at 6 weeks and 6 months of age (two-way ANOVA with Dunnett’s multiple comparison test, *N* = 12–14 per group) and **B**) Percentage change in CMAP amplitude between 6 weeks and 6 months of age in M102 dosed groups compared to vehicle (one-way ANOVA with Dunnett’s multiple comparison test, *N* = 12–14 per group). **C**) Change in repetitive stimulation amplitude between the 1st and 10th stimulus (one-way ANOVA with Dunnett’s multiple comparison test, *N* = 12–14 per group). **D**) Motor neuron counts from lumbar spinal cord in size bins (two-way ANOVA with Dunnett’s multiple comparison test, *N* = 5–6 per group). **E**) Cerebral cortex NRF2 and HSF1 targets after 3 months of dosing (two-way ANOVA with Dunnett’s multiple comparison test, *N* = 6 per group). All data shown as mean +/- SEM. Significance: * = *p* < 0.05, ** = *p* < 0.01, *** = *p* < 0.001, **** = *p* < 0.0001

Muscle electrophysiology was performed at 6 weeks and 6 months of age, where we have shown that there is a significant reduction in CMAP amplitude at 1, 3 and 6 months in earlier studies (see Suppl. Figure 2B) with an approximate 50% reduction in CMAP in *TDP-43*^*Q331K*^ mice compared to both non-transgenic littermates and *TDP-43*^*WT*^ transgenic mice. At 6 weeks of age there was no significant difference in compound muscle action potential (CMAP) amplitude between either of the M102 dose groups and the vehicle dosed group. However, at 6 months of age there was a significant improvement in CMAP amplitude in both M102 treated groups when compared to the vehicle group (Fig. 2A, two-way ANOVA with Dunnett’s post-test). The percentage change in CMAP amplitude between 6 weeks and 6 months of age also showed significant improvement of the M102 2.5 mg/kg BD dosed group when compared to the vehicle dosed group (Fig. 2B, one-way ANOVA with Dunnet’s post-test). At 6 months of age there was also a significant improvement in response to repetitive stimulation in the 2.5 mg/kg BD dosed animals and a similar numerical improvement in the 5 mg/kg OD group when compared to vehicle dosed animals, suggesting improved maintenance of the neuromuscular junctions (NMJs) (Fig. 2C, one-way ANOVA with Dunnett’s post-test).

Motor neuron counts and size distribution from the ventral horns of the lumbar spinal cord showed no significant difference between M102 dosed groups and vehicle dosed groups (Fig. 2D, two-way ANOVA with Dunnett’s post-test). However, in this model, we were unable to detect any significant difference between non transgenic (NT) and TDP-43^Q331K^ transgenic mice in lumbar spinal cord motor neuron counts at a similar timepoint (Suppl. Figure 2).

Analysis of RNA levels in the cerebral cortex by RT-qPCR showed an increase in downstream targets of NRF2 and HSF1 targets at 3 months of age in M102 dosed mice when compared to vehicle dosed mice, demonstrating activation of these pathways over time in the target tissue of the CNS (Fig. 2E, two-way ANOVA with Dunnett’s post-test). Finally, we assessed expression levels of TDP-43 by western blot to ensure M102 did not influence the overall expression level of TDP-43 (Suppl. Figures 2 C and 2D). No significant difference in TDP-43 protein levels were seen between any of the dose groups (*P* = 0.378, one-way ANOVA), confirming that the effects observed were not due to a reduction in TDP-43 expression.

### Pharmacokinetic data in wild-type mice (Suppl. Figure 3)

Following a single subcutaneous dose of 5 mg/kg in mice, M102 exhibited a mean Cmax (maximum plasma concentration) and AUC_last_ (area under the plasma concentration-time curve to the last measurable plasma concentration) of 989 ng/mL and 380 ng/mL*h, respectively. Pharmacokinetic (PK) evaluation of M102 post single dose oral administration was also performed. After a single oral dose of 10 mg/kg in mice, M102 showed a mean C_max_ and AUC_last_ of 168 ng/mL and 187 ng/mL*h, respectively. In the preclinical pharmacology studies in transgenic mouse models of ALS, M102 has demonstrated significant efficacy at a daily subcutaneous dose of 5 mg/kg [28]. Assuming a correlation of M102 efficacy to its systemic exposure and dose proportionality, the efficacious oral dose of M102 was estimated to be 25 mg/kg or lower.

### Oral administration of M102 in SOD1^G93A^ mice improves body weight, neurophysiology, and spinal cord motor neuron counts

We have previously shown disease modifying effects of M102 in the *SOD1*^G93A^ mouse model when M102 was dosed subcutaneously at 5 mg/kg daily [28]. In that study there was an upregulation of *Hmox1* and *Nqo1* in the spinal cord, a significant preservation of innervation at the neuromuscular junctions, a significant decrease in oxidised glutathione with an increase in the GSH/GSSG ratio in CNS tissue, and an improvement in rotarod performance and gait analysis parameters in the M102 exposed mice compared to the control group. We wanted to further expand on these data, by exploring the effect of M102 dosed orally at an equivalent dose to the subcutaneous dose, as this is the preferred route of administration in humans.

**Fig. 3:**
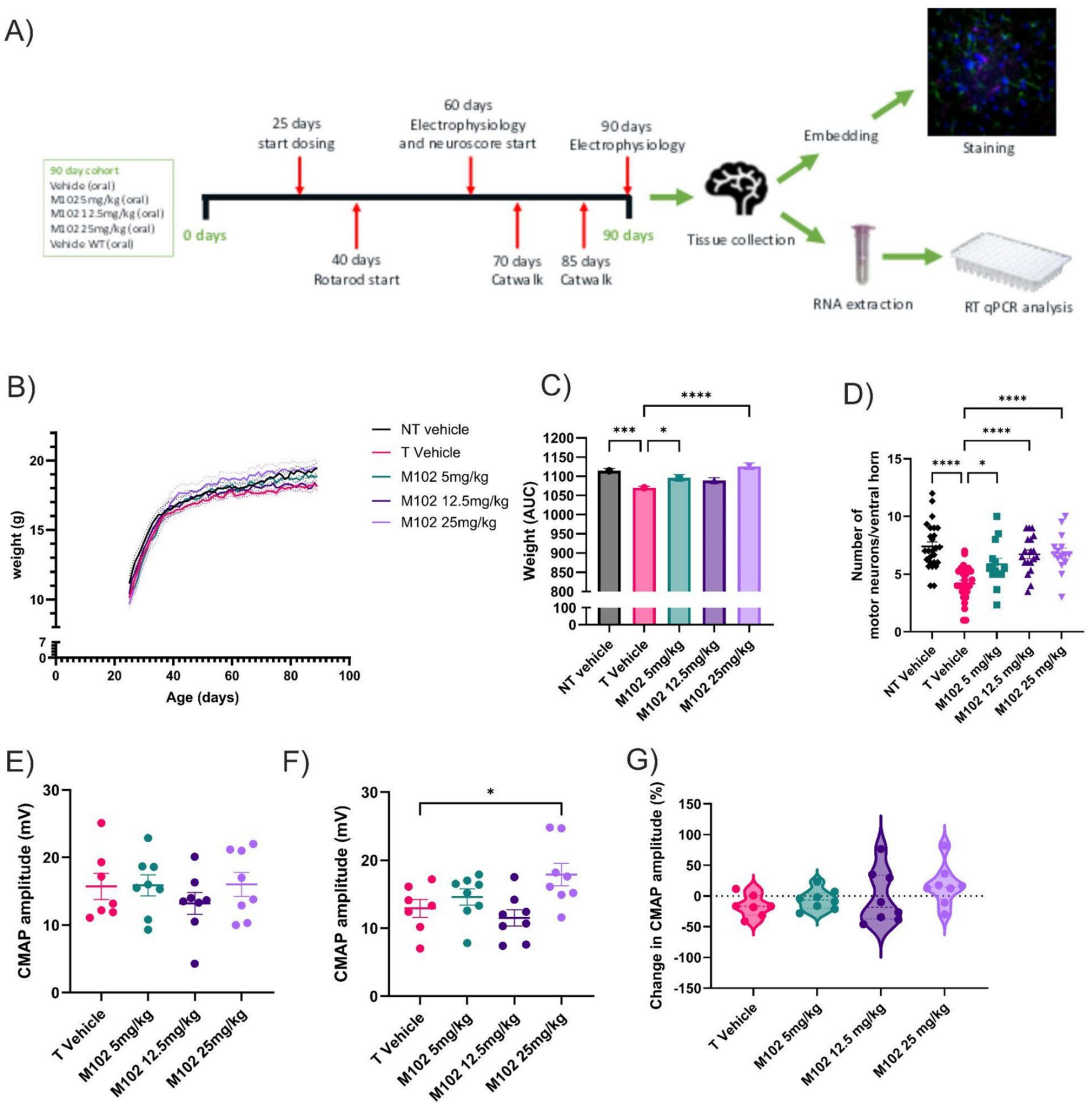
Oral M102 treatment in SOD1^G93A^ mice shows improvement in multiple parameters of disease progression. **A**) Design for the SOD1^G93A^ oral M102 study showing the main timepoints for electrophysiological and gait analysis. **B**) Body weights over time (two-way ANOVA with Dunnett’s multiple comparisons test. *N* = 8 per group. Dotted lines show +/- SEM). **C**) Area under the curve analysis of body weights (one-way ANOVA with Dunnett’s multiple comparison test, *N* = 8 per group). **D**) Motor neuron counts per ventral horn (one-way ANOVA with Dunnett’s multiple comparison test, *N* = 37–72 ventral horns per group). Compound muscle action potential (CMAP) amplitudes at **E**) 60 and **F**) 90 days of age (one-way ANOVA with Dunnett’s multiple comparison test, *N* = 7–8 per group). **G**) Percentage change in CMAP amplitude between 60 and 90 days of age (one-way ANOVA with Dunnett’s multiple comparison test, *N* = 7–8 per group). All data shown as mean +/- SEM. Significance: * = *p* < 0.05, ** = *p* < 0.01, *** = *p* < 0.001, **** = *p* < 0.0001

Transgenic female *SOD1*^G93A^ animals were block randomised into different groups and dosed orally with either vehicle, 5 mg/kg, 12.5 mg/kg or 25 mg/kg M102 daily from 25 days until 90 days of age (*n* = 8 per group). The doses were chosen as 5 mg/kg subcutaneous dosing has an equivalent exposure as 25 mg/kg oral dosing (Suppl. Figure 3). Behavioral tests were carried out at various time points to determine motor function and tissue was collected at 90 days for histological and mRNA analysis (Fig. 3A).

Animals were weighed daily before dosing to determine dose volume (Fig. 3B, two-way ANOVA followed by Dunnett’s post-test). *SOD1*^G93A^ transgenic mice are hypermetabolic and lose weight in the later stages of disease when compared to non-transgenic mice due to muscle and fat loss [37, 38]. Area under the curve analysis showed a significant increase in body weight, and thus improvement of disease progression, of the 5 mg/kg and 25 mg/kg M102 groups when compared to vehicle dosed animals (Fig. 3C; *p* < 0.05 at 5 mg/kg and < 0.001 at 25 mg/kg; one-way ANOVA followed by Dunnett’s post-test).

The number of motor neurons in the lumbar ventral horns was counted and a significant increase in the number of surviving motor neurons was observed in the M102 groups when compared to vehicle treated animals (Fig. 3D, one-way ANOVA with Dunnett’s post-test). At 90 days of age there was a significant improvement in the CMAP amplitude of the M102 25 mg/kg dose group when compared to vehicle controls (Fig. 3F, one-way ANOVA with Dunnett’s post-test). The percentage change in CMAP amplitude between 60 and 90 days of age showed a numerical increase in the M102 dosed groups when compared to the vehicle dosed group with 1/7 mice showing an increase in in CMAP in the vehicle group versus 6/8 in the 25 mg/kg M102 treated group (Fig. 3G, one-way ANOVA with Dunnett’s post-test).

### Dose prediction of M102 to humans and safety margin

Efficacy was observed in the mouse *SOD1*^G93A^ model at an oral dose of 12.5 mg/kg which resulted in a AUC_last_ of 419 ng*h/mL. Adjusting for the difference in human vs. mouse free fraction yields an estimated human equivalent exposure of 541 ng*h/mL. This value was used as the target efficacy exposure in the human dose projections as well as in the safety margin calculations. To confirm brain penetration of M102, additional plasma and brain PK studies were conducted in rats (Suppl. Table 1). After a single dose oral administration of 12 mg/kg M102, the brain-to-plasma ratios of M102 were determined to be 0.70 and 0.36 at 30 and 60 min post-dose, respectively. Subsequently, M102 was evaluated in non-Good Laboratory Practice (GLP) and GLP general toxicology studies in rats and non-human primates (NHPs) and the findings are summarised in Supplementary Table 2 (rats) and 3 (NHPs). In the non-GLP toxicology studies, M102-related mild liver toxicity findings were observed at 250 mg/kg and 100 mg/kg in rats and NHPs, respectively. In 28-day GLP toxicology studies, 75 mg/kg was declared as the no observed adverse effect level (NOAEL) in both rats and non-human primates (NHPs). The free AUC on Day 28 at the NOAEL in both species, as well as the margin to the projected human free AUC necessary for efficacy, is 9.3-fold for rats, 10.1-fold for female NHPs, and 15.1-fold for male NHPs.

Human clearance, volume of distribution and half-life were estimated using PK data from rat and monkey studies. Various allometric scaling techniques were applied following the rule of exponents guidelines. Human clearance was also estimated from in vitro hepatocyte incubations. Correcting for brain weight, free fraction and using the multi-exponential method [39] resulted in estimates ranging from 55 to 111.7 mL/min/kg, while scaling intrinsic clearance from human hepatocytes resulted in an estimate of 84 mL/min/kg. Human volume of distribution was estimated by allometric scaling of preclinical species values and using an exponent of 1 and was approximately 81 L/kg. M102 has a bioavailability range of 10–30% in preclinical species. As a predictive model of human bioavailability does not exist, this range of potential values were modelled. Using the projected human efficacious exposure target of 302 ng*h/mL that was based on mouse efficacy at an oral dose of 12.5 mg/kg PO, a daily dose range in humans of 352–1417 mg is predicted.

### Increased oxidised RNA in affected CNS areas, and in the CSF and i-Astrocytes derived from ALS cases

Encouraged by the compelling data obtained in two in vivo models of ALS, we decided to evaluate the levels of oxidative stress detectable in patient biosamples and in a patient-derived cellular model of ALS, as potential biomarkers of target engagement and efficacy.

**Fig. 4:**
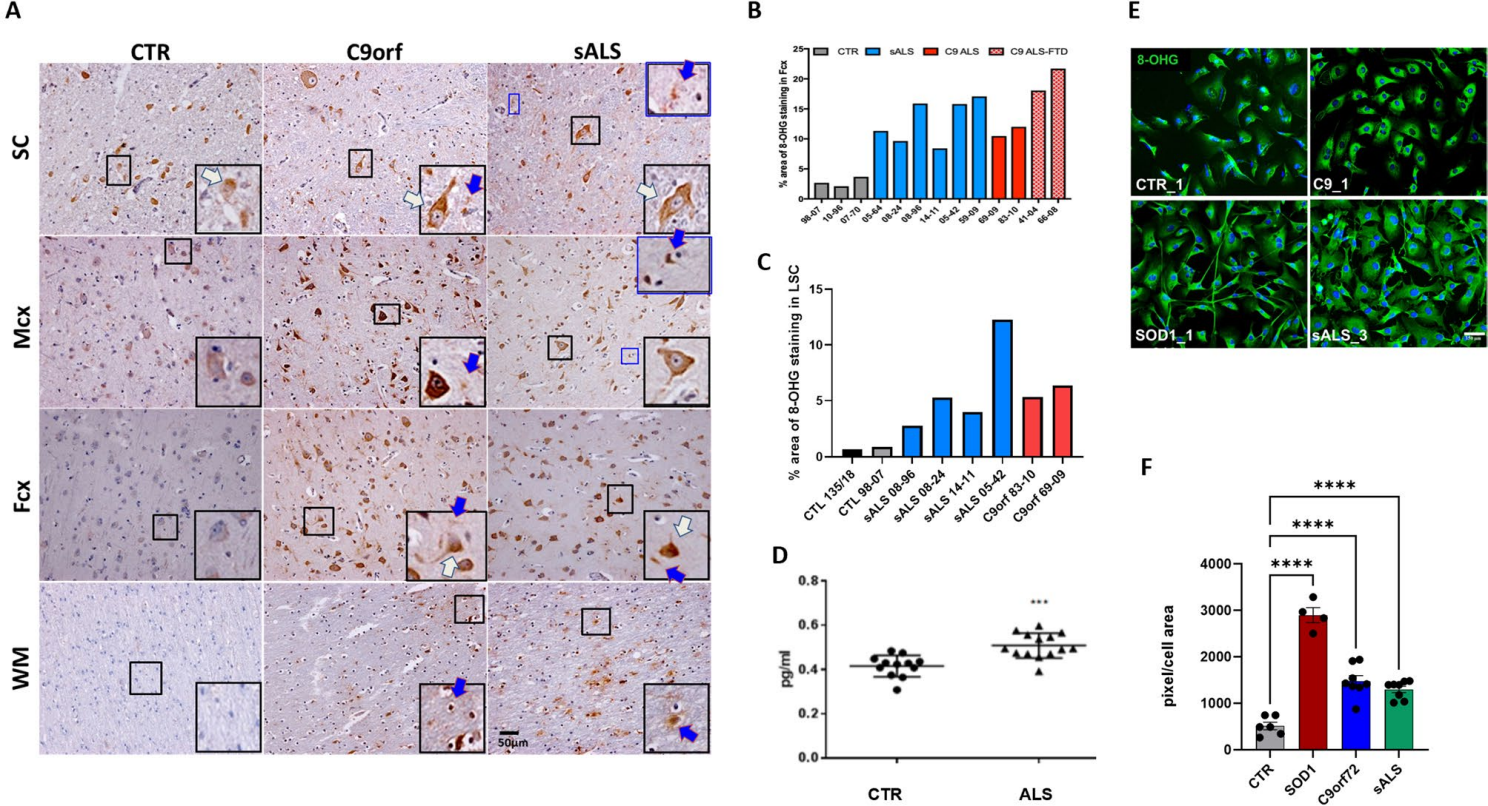
ALS cases display high levels of oxidised RNA compared to healthy controls. **A**) Representative images of 8-OHG staining in post-mortem tissue show increased oxidised RNA levels in neurons (white arrows) and non-neuronal cells (blue arrows) in C9-ALS and sALS patients compared to healthy controls, in several areas of the CNS. **B**) Respective quantifications in the frontal cortex and **C**) lower spinal cord (each bar represents one individual). **D**) Increased levels of oxidative stress are also detected in the CSF of ALS cases compared to healthy controls (*N* = 13 ALS and *N* = 12 controls). Unpaired t-test. **E**) Representative images of 8-OHG staining in iAstrocytes derived from healthy controls and *SOD1*-, *C9*orf72- and sporadic ALS cases (scale bar: 150 μm), and F) Respective quantifications show that patient-derived iAstrocytes also display high levels of oxidative stress, recapitulating what is observed in post-mortem tissue (*N* = 3 controls; *N* = 1 *SOD1*, *N* = 3 *C9*-ALS; *N* = 3 sALS). One-way ANOVA followed by Dunnett’s multiple comparisons test. SC: spinal cord; LSC: lower spinal cord; Mcx: motor cortex; Fcx: frontal cortex; WM: white matter. All data shown as mean +/- SEM. Significance: * <0.05; ** <0.01; *** <0.001, ****<0.0001

Oxidative stress is one of the hallmarks of ALS, with oxidised lipids and proteins being identified in post- mortem tissues [40]. Due to the recently identified involvement of RNA dysregulation in ALS [41] and the known role for oxidised RNA in neurodegeneration [42], we aimed to map the presence of oxidised RNA in the areas of the CNS that are affected by the disease. As guanine is the base that is most susceptible to oxidation, we used an antibody against 8-oxo-2-oxyguanosine (8-OHG), as a marker of direct nucleic acid oxidation. Staining of frontal and motor cortex with adjacent white matter, as well as the cervical spinal cord, from 6 sALS patients, 4 patients carrying *C9orf72* repeat expansion mutations and 3 healthy controls (subject information in Suppl. Table 4) revealed that ALS patients display much higher levels of oxidised RNA in neurons and non-neuronal cells compared to age-matched, neurologically unaffected controls (Fig. 4A-C). Interestingly, although ALS patients displayed higher levels of oxidised RNA in the spinal cord compared to controls (Fig. 4C), amongst all the CNS areas analysed, the spinal cord was the tissue with the highest oxidative stress burden (Fig. 4A).

Given the positive correlation between 8-OHG staining and pathology, we proceeded to examine whether oxidised RNA levels in the CSF could be used as a biomarker of oxidative stress. Using an ELISA kit specific for oxidised RNA, we detected higher levels of 8-OHG in the CSF of ALS patients (*n* = 13; 9 males and 4 females; average age = 56.2 ± 10 years) compared to controls (*n* = 12; 5 males and 7 females; average age = 44.2 ± 12 years (Fig. 4D, unpaired t-test). However, there did not seem to be any correlation between clinical/genetic characteristics and levels of oxidised RNA (clinical information in Suppl. Table 5). Furthermore, the levels of oxidised RNA did not correlate with age in healthy controls, suggesting the higher levels of oxidised RNA identified in the ALS cases are not due to a potential age difference (Suppl. Figure 4).

Astrocytes serve as the primary regulators of the oxidative stress response in the CNS, owing to their robust antioxidant capacity and efficient activation of the NRF2 signalling pathway [43]. They play a critical neuroprotective role by buffering reactive oxygen species (ROS) and supplying key antioxidants to support both neurons and other glial cell types. To determine whether increased levels of oxidised RNA were also present in ALS patient-derived astrocytes in vitro, we stained iAstrocytes differentiated from induced neural progenitor cells (iNPCs) directly reprogrammed from fibroblasts collected via skin biopsy from ALS cases and age-matched healthy controls (patient information in Suppl. Table 6). These iAstrocytes are known to retain the features of ageing that are likely to contribute to patient-specific phenotypes [44], and therefore constitute a robust in vitro model of the astrocyte contribution to ALS. iAstrocytes derived from *SOD1*, *C9orf72* and sporadic ALS patients displayed higher levels of oxidised RNA compared to healthy controls (Fig. 4E, F, one-way ANOVA followed by Dunnett’s multiple comparisons test), recapitulating the findings in post-mortem tissue.

### M102 treatment is a dual activator of the NRF2 and HSF1 pathways in ALS patient-derived iAstrocytes, and is sufficient to reduce oxidative stress, misfolded SOD1, and TDP-43 proteinopathy in vitro

As ALS patient-derived iAstrocytes recapitulate the high levels of oxidative stress observed in post-mortem tissue, we set out to evaluate the ability of M102 treatment to activate the antioxidant NRF2 pathway and therefore contribute to decreased oxidative stress in these cells.

**Fig. 5:**
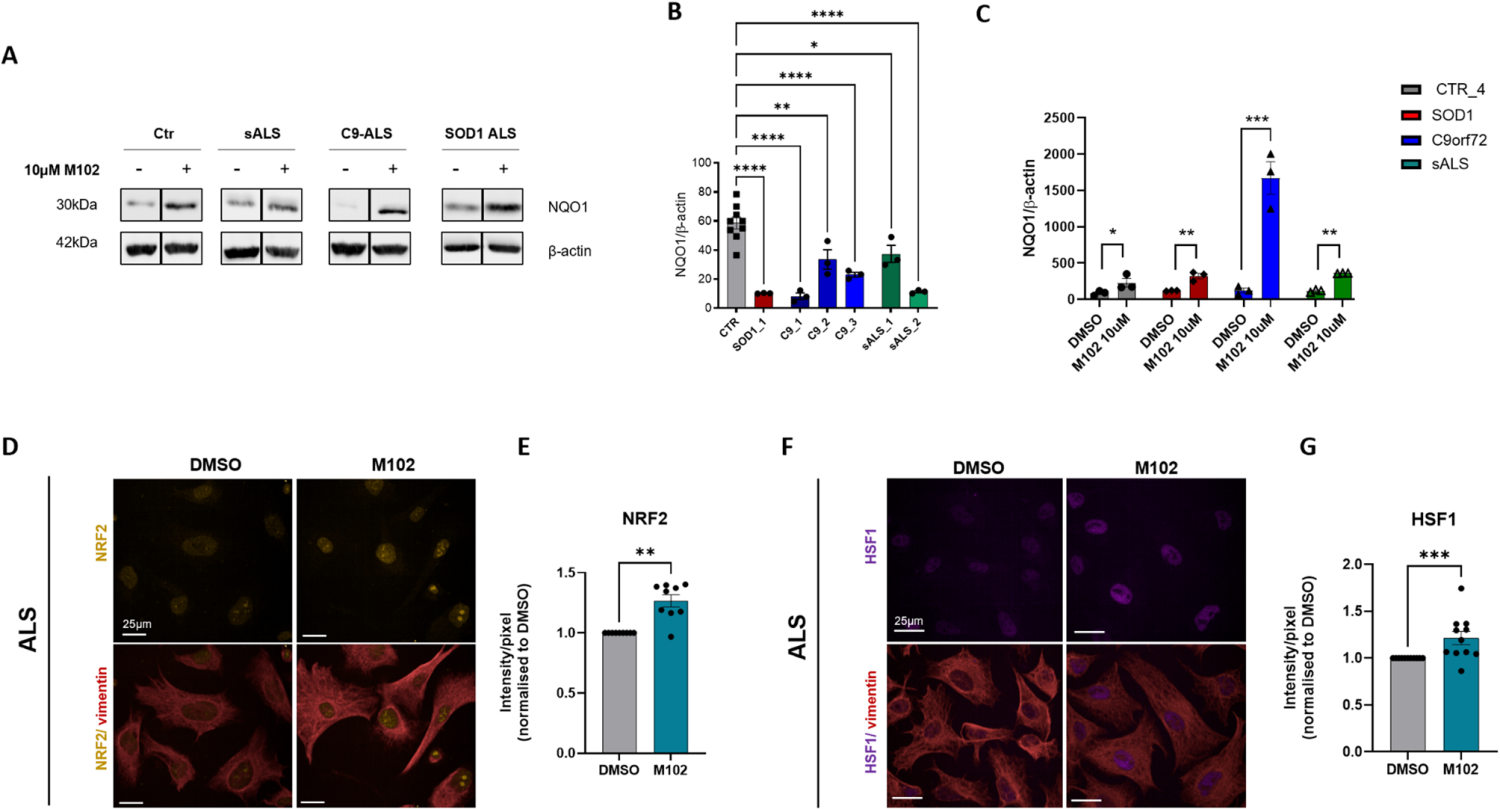
M102 treatment activates both NRF2 and HSF1 pathways in patient-derived iAstrocytes. **A**) Representative western blots showing increased expression of NQO1 upon 10 µM M102 treatment for 48 h in sporadic, *C9* and *SOD1* ALS patient-derived iAstrocytes. DMSO and M102 treated samples of each line are from the same blot but were cropped because there were different conditions in between these lanes. Original full blots shown in Suppl Fig. 5. **B**) Quantification shows that iAstrocytes derived from patients with different genotypes display lower baseline levels of NQO1 compared to healthy controls (*N* = 3 technical repeats per cell line; one-way ANOVA followed by Dunnett’s multiple comparisons test), and **C**) these levels are increased upon 10 µM M102 treatment for 48 h (*N* = 3 technical repeats per cell line; two-way ANOVA followed by Šídák’s multiple comparisons test). **D**) Representative staining of NRF2 before and after 10 µM M102 treatment in ALS iAstrocytes, and respective quantifications under **E**) Baseline conditions and in response to M102 treatment for 24–48 h. **F**) Representative staining of HSF1 before and after 10 µM M102 treatment in sALS and C9-ALS iAstrocytes, and respective quantifications under G) Baseline conditions and upon M102 treatment for 24–48 h.E), **G**) 2 sALS and 2 C9-ALS lines, 2–3 technical repeats each. Each dot represents a technical repeat. Unpaired t-test. All data shown as mean +/- SEM. Significance: * <0.05; ** <0.01; *** <0.001

It is known that the NRF2 pathway is downregulated in astrocytes from models of ALS [27]. Consistently, iAstrocytes derived from *SOD1* (*n* = 1), *C9orf72* (*n* = 3) and sporadic (*n* = 2) ALS cases displayed lower levels of NQO1, a downstream target of NRF2 [18, 20], compared to age-matched healthy controls, under baseline conditions (Fig. 5A, B, one-way ANOVA followed by Dunnett’s multiple comparisons test; full blots in Suppl. Figure 5). Importantly, when treated with 10 µM of M102 for 48 h, the levels of NQO1 increase significantly in iAstrocytes from healthy controls and ALS patients, including *SOD1*, *C9orf72* and sporadic patient lines (Fig. 5A, C, two-way ANOVA followed by Šídák’s multiple comparisons test). Overall NRF2 levels in the nucleus vs. cytoplasm also increased upon 24–48 h treatment with M102 (Fig. 5D, E, unpaired t-test), providing evidence that M102 activates the NRF2-ARE pathway in ALS patient-derived iAstrocytes. Similar to what is observed in mouse models of ALS, M102 treatment also leads to increased expression of HSF1 in patient-derived iAstrocytes (Fig. 5F, G, unpaired t-test), indicating that M102 is a dual activator of the NRF2-ARE and HFS1-HSE pathways in vitro.

**Fig. 6:**
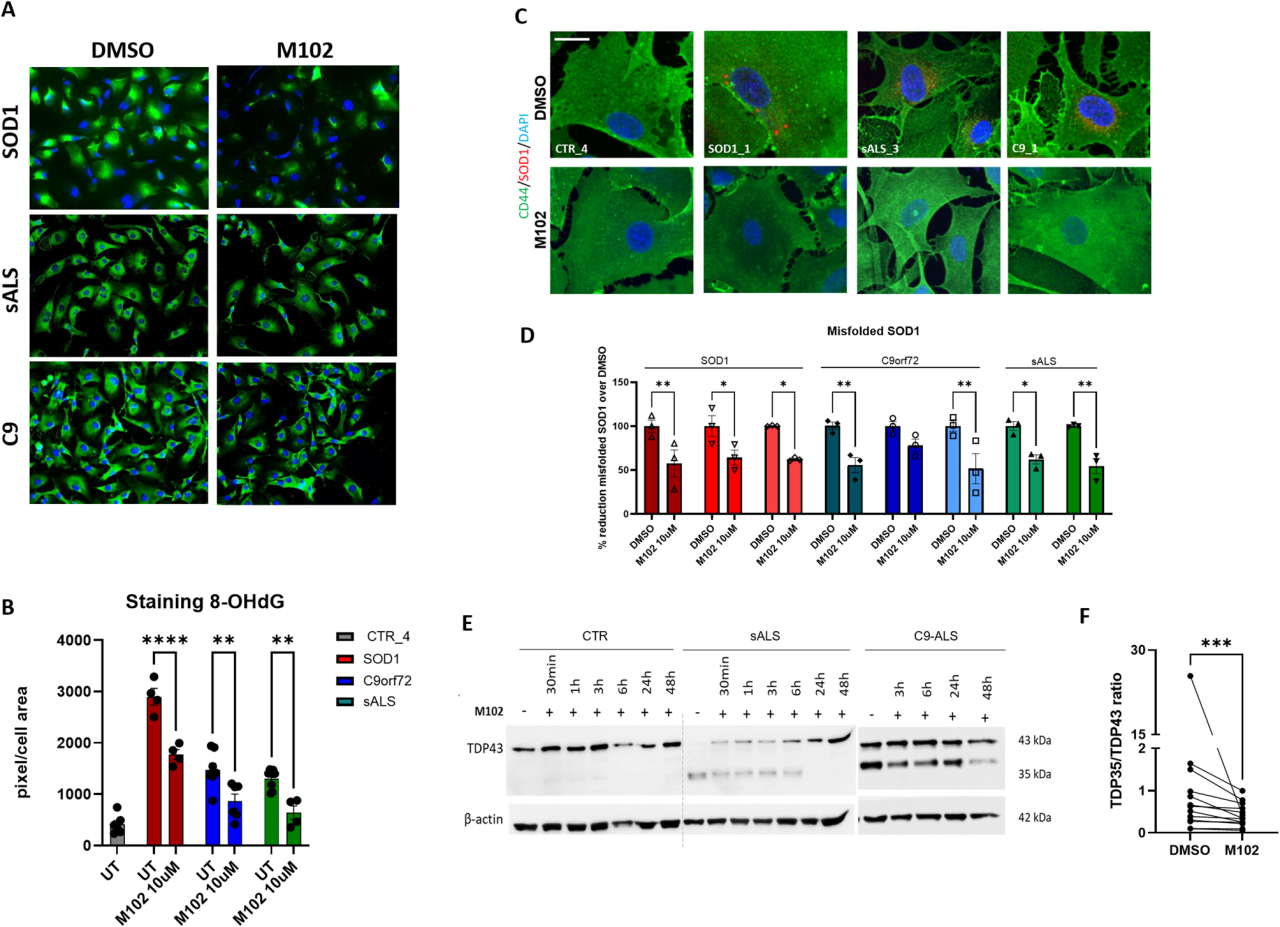
M102 treatment reduces oxidised RNA, misfolded SOD1 and TDP-43 proteinopathy in ALS patient-derived iAstrocytes. **A**) Representative images of 8-OHdG staining and (**B**) respective quantifications show reduced levels of oxidised RNA upon 10 µM M102 treatment for 48 h in iAstrocytes derived from *SOD1*, sporadic and *C9* ALS patients. *N* = 2–3 per genotype. Two-way ANOVA followed by Šídák’s multiple comparisons test. **C**) Representative images of misfolded SOD1 before (top panel) and after (bottom panel) exposure to M102 and **D**) respective quantifications show reduction of misfolded SOD1 in iAstrocytes derived from *SOD1*, sporadic and *C9* ALS patients upon M102 treatment (*N* = 3 *SOD1* ALS, *N* = 3 *C9*-ALS, *N* = 2 sALS - each with 3 technical repeats). Two-way ANOVA followed by Šídák’s multiple comparisons test. **E**) Representative immunoblotting images and **F**) Respective quantifications show a time-dependent reduction of TDP-43 proteinopathy upon M102 treatment (10 μm for 48 h) in sALS (*n* = 10, 2–4 technical repeats each) and *C9*-ALS (*n* = 3, 3–4 technical repeats each; paired t-test) patient-derived iAstrocytes, characterised by a reduction of the fragmented 35 kDa band. All data shown as mean +/- SEM. Significance: * <0.05; ** <0.01; *** <0.001

Consistent with these findings, iAstrocytes derived from *SOD1*, *C9orf72* and sporadic ALS patients treated with 10 µM M102 for 48 h showed decreased levels of oxidised RNA, thus confirming that M102 is capable of reducing the levels of oxidative stress in ALS astrocytes (Fig. 6A, B, two-way ANOVA followed by Šídák’s multiple comparisons test).

The HSF1-HSE pathway is known to play an essential role in maintaining proteostasis by facilitating protein folding and avoiding protein misfolding [35]. As we showed that M102 is a strong activator of the HSF1 pathway, we set out to investigate whether M102 treatment has a beneficial effect on the reduction of misfolded SOD1 and TDP-43 proteinopathy, two hallmarks of ALS pathology [45, 46]. By using an antibody raised against misfolded SOD1 (B8H10), we detected perinuclear staining in iAstrocytes from *SOD1* cases, as well as cases carrying *C9orf72* mutations and sALS patients, consistent with previous findings in post-mortem tissues [47]. Upon treatment with M102, we observed a significant reduction of misfolded SOD1 in iAstrocytes derived from *SOD1*,* C9orf72*, and sporadic ALS patients (Fig. 6C, D, two-way ANOVA followed by Šídák’s multiple comparisons test). Induced astrocytes recapitulate one of the key hallmarks of ALS, i.e. TDP-43 proteinopathy, detected as the presence of TDP-43 fragments (observed at 35 kDa) in sporadic and *C9orf72* ALS patient iAstrocytes (Fig. 6E), but not in *SOD1* cases (Suppl. Figure 6). Importantly, we observed that time-dependent treatment with M102 leads to a reduction of TDP-43 proteinopathy, particularly upon 48 h exposure (Fig. 6E, F, paired t-test).

We next analysed the effect of M102 treatment on TDP-43-regulated cryptic splicing by qRT-PCR. *ATG4B *and *EXD3* - two TDP-43 target genes with previously reported cryptic splicing events [48, 49] and significant astrocytic expression (67.9 and 48.8 nTPM, respectively; https://www.proteinatlas.org/) - were selected. Notably, *EXD3* is primarily expressed in astrocytes and shows low neuronal expression (https://www.proteinatlas.org/). Despite significant variability between cell lines, we observed a trend towards higher levels of cryptic exon inclusion in sALS lines for these targets (Suppl. Figure 7 A; Mann-Whitney U test). M102 treatment led to a significant decrease in the cryptic splicing levels in sALS lines. Interestingly, the two genes displayed different temporal dynamics, where *ATG4B* responded early (48 h) and *EXD3* responded at later time-points (72–96 h) (Suppl. Figure 7B; Kruskal-Wallis test followed by multiple comparisons).

### M102 rescues MN survival in co-culture with ALS patient-derived iAstrocytes by targeting multiple mechanisms known to underlie ALS pathophysiology

**Fig. 7:**
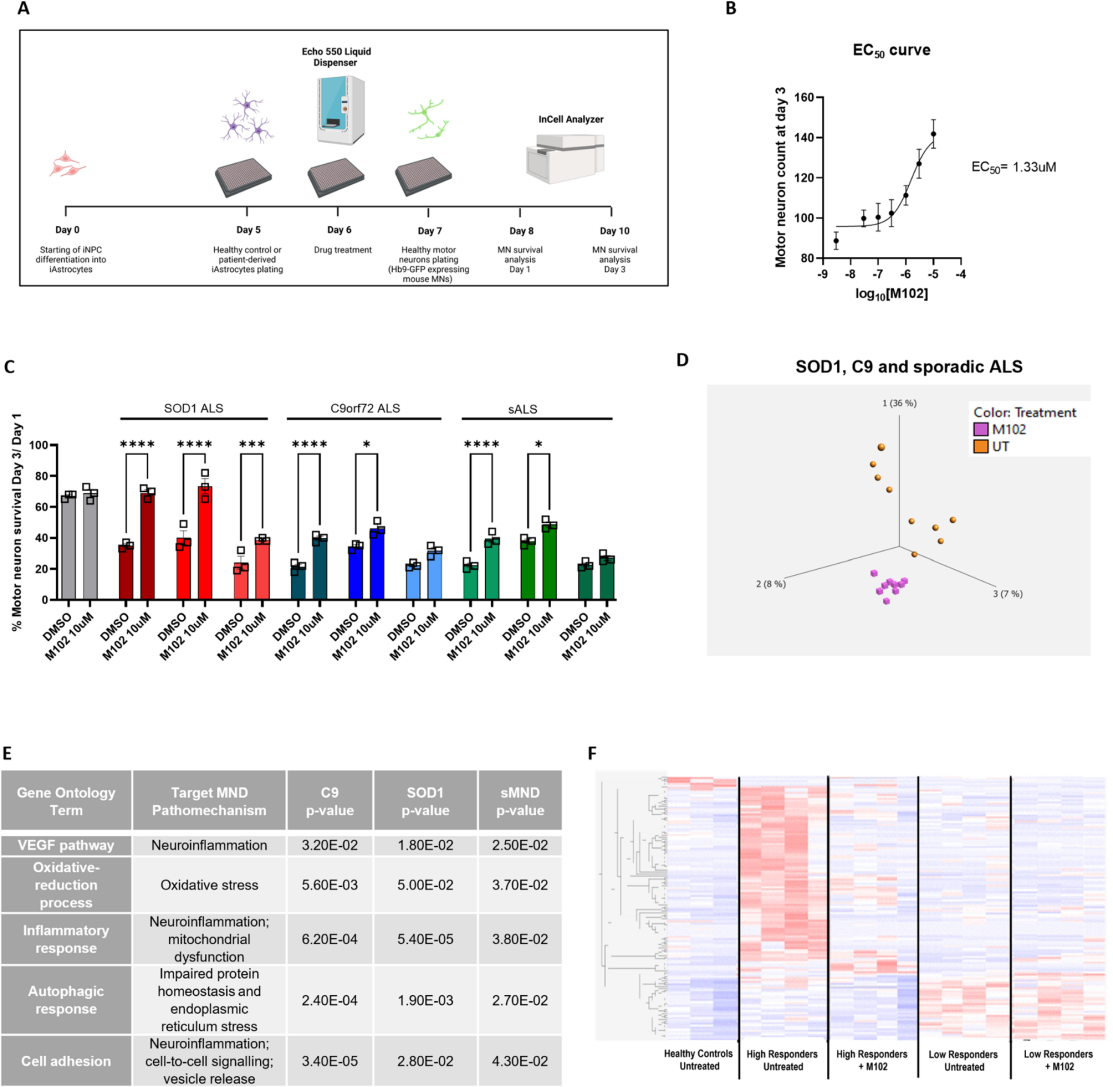
M102 treatment rescues MN survival in co-cultures with *C9*, *SOD1* and sporadic ALS patient-derived astrocytes. **A**) Schematic figure of the MN-iAstrocyte co-culture system previously described [74]. **B**) EC50 calculated from dose-response curve tested in sALS patient-derived iAstrocyte/MN co-cultures (average of 4 cell lines, 6–8 technical repeats each). **C**) ALS-patient derived iAstrocytes are toxic to co-cultured MNs under basal conditions, leading to increased MN death compared to co-cultures with astrocytes from healthy controls, and motor neuron survival is rescued upon 10 µM M102 treatment for 48 h in sporadic, *C9* and *SOD1* ALS cases (*N* = 3 for each subtype). Two-way ANOVA followed by Šídák’s multiple comparisons test. **D**) RNA sequencing from iAstrocytes treated with 10 µM M102 or DMSO for 48 h reveal transcriptomic changes in response to M102 treatment in ALS iAstrocytes. **E**) Enriched pathways in response to M102 treatment identified in common between *C9*,* SOD1* and sALS iAstrocytes show that M102 treatment targets various pathophysiological mechanisms known to play a role in ALS. **F**) Heatmap shows the transcriptomic profile of high and low responders (i.e. >50% rescue and < 50% rescue, respectively) to M102 under baseline conditions and upon treatment with 10 µM M102 for 48 h. All data shown as mean +/- SEM. Significance: * <0.05; ** <0.01; *** <0.001

Astrocytes from ALS patients are known to be toxic to MNs, contributing to death of healthy motor neurons in co-culture [10]. We have previously reported that iAstrocytes differentiated from iNPCs directly reprogrammed from fibroblasts of *SOD1*,* C9orf72* and sporadic ALS patients are toxic to MNs [10]. As M102 seems to target multiple mechanisms associated with ALS, including oxidative stress and protein misfolding and aggregation, we asked whether M102 treatment would be sufficient to rescue MN survival when in co-culture with toxic ALS astrocytes. For this, we used our previously described iAstrocyte-MN co-culture model [10]. Briefly, ALS patient-derived iAstrocytes were treated with DMSO or M102, using the Echo 550 liquid dispenser, and 24 h later co-cultured with healthy mouse MNs expressing GFP under a Hb9 promoter. The MNs were then scanned in an InCell Analyser 24 h and 72 h after treatment, and the numbers of viable MNs counted (Fig. 7A).

To assess the optimal dose of M102 to use, we started by testing 6 different concentrations of M102, ranging from 0.03 µM to 10 µM, in 4 sALS iAstrocyte lines. As expected, under baseline conditions, MNs co-cultured with ALS iAstrocytes showed reduced survival compared to MNs co-cultured with iAstrocytes derived from healthy controls. Furthermore, our dose-curve response showed that M102 has an EC_50_ of 1.33 µM and the maximum neuroprotective effect in co-culture was achieved with a dose of 10 µM (Fig. 7B). We therefore used 10 µM M102 to further evaluate whether M102 is sufficient to rescue MN survival when in co-culture with multiple iAstrocyte lines derived from both familial and sporadic ALS cases. Under baseline conditions, MNs co-cultured with ALS patient-derived iAstrocytes displayed reduced survival (between 30% and 60% reduction in MN survival depending on the patient donor) compared to MNs co-cultured with iAstrocytes derived from healthy controls (Fig. 7C). Treatment with 10 µM M102 led to a significant increase in MN survival in co-cultures with 7 out of 9 different iAstrocyte patient lines, including *SOD1*,* C9orf72* and sporadic ALS cases (Fig. 7C, two-way ANOVA followed by Šídák’s multiple comparisons test). Interestingly, the response to M102 varied between patient lines, with 4 patient lines responding more strongly by increasing MN survival by > 50% compared to baseline levels.

In order to further explore the effects of M102 and better understand how M102 could play a role in iAstrocyte toxicity and MN survival, we performed RNA Sequencing in patient-derived iAstrocytes before and after 10 µM M102 treatment for 48 h. Our data show that there is a clear separation of the transcriptomic profile before and after M102 treatment, as shown by a principal component analysis (PCA) plot (Fig. 7D). This transcriptomic shift in response to M102 is observed in iAstrocytes derived from *SOD1*,* C9orf72*, and sporadic patients (Suppl. Figure 8 A, B,C, respectively). Differential expression analysis between treated and untreated iAstrocytes identified a total of 160, 283, and 267 differentially expressed genes (DEGs) in *C9-*ALS cases, *SOD1* cases, and sALS cases, respectively (Suppl. Figure 8D). We further performed gene ontology analysis to investigate the predominant pathways affected by M102 treatment. The results show that M102 treatment altered the expression of genes associated with neuroinflammation, mitochondrial dysfunction, autophagic response and cell adhesion (Fig. 7E, Suppl. Table 7), all known to be important drivers of the pathophysiology of ALS [9]. Taken together with our results demonstrating that M102 is a dual activator of the NRF2 and HSF1 pathways, these results indicate that M102 targets multiple pathophysiological mechanisms operating in ALS.

As we observed that some iAstrocyte lines responded more strongly to M102 than others, we then set out to test whether we could discriminate high and low responders to M102 based on the individual patient transcriptomic profiles. For this, we classified the iAstrocytes in ‘high’ and ‘low’ responders to M102 based on the levels of motor neuron survival upon M102 treatment in iAstrocyte-MN co-cultures. iAstrocytes that responded to M102 (p-value < 0.05 in M102 vs. DMSO) with an increase in motor neuron survival of over 50% (compared to DMSO) were considered ‘high responders’ whereas the iAstrocytes that responded with an increase in motor neuron survival less than 50% were considered ‘low responders’. Following these criteria, 5 patients were considered high responders and 4 were considered low responders (see Suppl. Table 8). Due to poor RNA quality, one SOD1 patient responder was not included in the RNA-sequencing analysis, thus leaving 4 high responders. A heatmap of healthy controls and ALS patient-derived iAstrocytes before and after M102 treatment showed a set of 161 transcripts identified significant differences between “high” responders to M102 before and after treatment. Interestingly, upon M102 treatment, high responders displayed a shift in their transcriptomic profile for these 161 transcripts, which became more similar to the profile of healthy controls (Fig. 7F), thus indicating that a subgroup of these genes could be used as a biomarker of drug response. In contrast, minimal transcriptional changes were observed in these 161 transcripts in the “low responders”.

## Materials and methods (For full materials and methods, see Supplementary Material)

### Study design

This study aims to provide a comprehensive package of preclinical efficacy data for M102, a CNS penetrant small molecule electrophile capable of activating both NRF2-ARE and HSF-1-HSE pathways, in vivo and in vitro. To do this, the pharmacokinetic profile was assessed in C57Bl/6 mice; GLP general toxicology studies were performed in rats and NHPs; and oral dose, target engagement and efficacy were assessed across two ALS mouse models: *TDP-43*^Q331K^ and *SOD1*^G93A^. RT-qPCR was used to assess target engagement after M102 treatment. Immunohistochemistry (IHC) was used to identify and count the number of motor neurons in the spinal ventral horn after M102 treatment. Compound muscle action potential (CMAP) amplitude of hind limb muscles, rotarod performance, and gait parameters were used to assess treatment efficacy on readouts of motor function. Together, these data enabled prediction of human efficacious exposures and doses, which were established to be well within the safety margin predicted from GLP toxicology studies.

Post-mortem tissue, human CSF samples, and ALS patient-derived astrocytes were used to confirm that ALS patients present higher levels of oxidative stress compared to healthy controls, and that M102 is capable of reducing the levels of oxidative stress. A combination of immunocytochemistry (ICC) and western blotting analysis was used to assess the effects of M102 on NRF2-ARE and HSF-1-HSE pathway activation, as well as to assess TDP-43 proteinopathy in patient-derived astrocytes. MN-astrocyte co-cultures were performed to assess whether M102 can rescue motor neuron survival in the presence of toxic ALS iAstrocytes, and to determine high and low responders to M102. RNA Sequencing of untreated and M102 treated patient-derived iAstrocytes was used to identify additional biological mechanisms targeted by M102.

No study size calculations or randomization were carried out. All samples were quantified in a blinded manner for microscopy and mouse experiments. No cell or animal samples were excluded.

### Ethics statement

GLP general toxicology studies in rats and NHPs to evaluate potential toxicity of M102 were conducted at WuXi AppTec (Suzhou, China). The protocol and any amendments or procedures involving the care or use of animals in this study were reviewed and approved by the Institutional Animal Care and Use Committee (IACUC) prior to the initiation of such procedures. A staff veterinarian monitored the study for animal welfare issues.

All mouse studies were carried out under a UK Home Office project license by individuals that held the appropriate UK Home Office personal license and had appropriate training for procedures. All work was carried out under the terms of the UK Animals (Scientific Procedures) Act 1986 and animals were housed and maintained in line with Home Office Code of Practice for House and Care of Animals Used in Scientific Procedures.

Formalin-fixed, paraffin-embedded (FFPE) human CNS post-mortem tissue was obtained from the Sheffield Brain Tissue Bank with Research Ethics Committee approval (Sheffield Brain Bank –SBB-, Ethics Committee reference 08/MRE00/103). Human CSF was obtained from the University of Sheffield Biorepository with Research Ethics Committee approval number STH16573. Fibroblasts were collected from skin biopsies donated by ALS patients and controls with informed consent (Ethical Committee approval references: 12/YH/0330; 16/LO/2136).

### Mice

Mice were housed in same sex groups of between 2 and 5 mice per cage. Each cage consisted of a plastic house, sawdust covering the floor (Datesand) and paper wool bedding (Datesand). The mice had ad libitum access to water and food (standard rodent diet 2018, Envigo). Temperatures in the rooms were maintained at 21 °C with a 12 h light/dark cycle (7am – 7pm). Wild type animals used were C67BL/6 mice either from Envigo or non transgenic mice from the SOD1^G93A^ colony.

The *SOD1*^G93A^ C57BL/6 transgenic mice were bred in-house. The B6SJL-Tg(*SOD1*-G93A)1Gur/J mice were backcrossed onto the C57BL/6J OlaHsd background for at least 20 generations. This line has been extensively characterised in-house and develops a reproducible progressive motor phenotype [50]. The *TDP-43*^Q331K^ C57BL/6NJ transgenic mice were bred in-house and were obtained from Jackson laboratory (stock number 017933). These mice were originally on a C57BL/6NCrl background [51] but have been crossed onto a C57BL/6NJ background for a minimum of 4 generations and have been extensively characterised by the authors [32]. Mice were ear-clipped for identification and genotyping. Genotyping for the two colonies was carried out as previously described [32, 50].

### Differentiation of patient-derived neurons, motor neurons and astrocytes

#### Tissue culture to generate induced neural progenitor cells (iNPCs) and iAstrocytes from donated fibroblasts

Fibroblasts collected from skin biopsy material donated by ALS patients and controls were directly reprogrammed into iNPCs, using a combination of retroviral vectors (Oct3/4, Sox2, Klf4, and c-Myc), as previously described [10]. Briefly, the fibroblasts were treated with 700 µL medium/viral vector overnight, then washed 2× with PBS, and fed once per day with fibroblast medium (DMEM plus 10% FBS) for 3 days. At day 4 the cells were switched to NPC conversion medium consisting of DMEM/F12, 1% N2, 1% B27, 20 ng/mL FGF2, 20 ng/mL EGF, and heparin (5 µg/mL; Sigma-Aldrich) and fed every day thereafter. When the cells changed shape and presented sphere-like structures, they were lifted with accutase, centrifuged, resuspended in NPC conversion medium, and replated for expansion. Once the NPC culture was established, the medium was switched to NPC medium consisting of DMEM/F12, 1% N2, 1% B27, and FGF2 (40 ng/mL). To differentiate iNPCs into iAstrocytes, iNPCs were seeded in NPC medium at low density in a fibronectin-coated 10-cm dish. The day after, the medium was changed to DMEM containing 10% FBS and 0.3% N2 and the cells were allowed to mature for at least 7 days.

#### Motor neuron differentiation from embryonic stem cells

Mouse embryonic stem cells expressing GFP under the MN-specific promoter HB9 (HBG3 cells; kind gift from Tom Jessell, Columbia University, New York) were cultured on primary mouse embryonic fibroblasts (Millipore). For differentiation into MNs, cells were lifted with trypsin and resuspended in DFK10 culture medium consisting of knockout DMEM/F12, 10% knockout serum replacement, 1% N2, 0.5% L-glutamine, 0.5% glucose (30% in water), and 0.0016% 2-mercaptoethanol. The cells were plated on nonadherent Petri dishes to allow formation of embryoid bodies. After 1 d of recovery, 2 µM retinoic acid (Sigma) and 1 µM smoothened antagonist (SAG, Merk) were added freshly every day with fresh medium. After 5 days of differentiation, the embryoid bodies were dissociated and sorted for GFP on a BD FACSVantage/DiVa sorter.

#### Human iAstrocyte-murine motor neuron co-culture assay

Human plasma fibronectin (Merck Millipore) was diluted 1:400 in PBS, and 5 µL was added per well on 384-well plates (Greiner Bio-one, 781091. Plates were coated for at least 5 min at RT. A total of 2,000 human iAstrocytes were seeded in 35 µL iAstrocyte media per well on fibronectin-coated 384-well plates. Plates were centrifuged at 1,760 x g for 60 s, and cells were incubated for 24 h. Drugs were then delivered to iAstrocytes in 100% anhydrous DMSO (Sigma, 276855) using an Echo550 liquid handler (Labcyte). The final concentration of DMSO was 0.1% (v/v) in the media in all wells. Plates were centrifuged at 1,760 x g for 60 s, and cells were incubated for a further 24 h. A total of 2,500 murine Hb9-GFP + motor neurons were seeded per well in motor neuron media (KnockOut DMEM (45% v/v), F12 medium (45% v/v), KO Serum Replacement (10% v/v), 50 units/ml penicillin/streptomycin (Lonza), 1 mM L-glutamine, 1X N-2 supplement (Thermo-Fisher Scientific), 0.15% filtered glucose, 0.0008% (v/v) 2-mercaptoethanol, 20 ng/ml GDNF, 20 ng/ml BDNF, 20 ng/ml CNTF) on top of the pre-treated iAstrocytes. Plates were centrifuged at 1,760 x g for 60 s. Hb9-GFP + motor neurons were imaged after 24 and 72 h using an INCELL analyser 2000 (GE Healthcare), and the number of viable motor neurons was counted using the Columbus™ analysis software (Perkin Elmer). The number of viable motor neurons (defined as GFP + motor neurons with at least 1 process) that survived after 72 h in co-culture was calculated as a percentage of the number of viable motor neurons after 24 h in co-culture. The percentage survival of motor neurons was then normalised to the DMSO control for each individual iAstrocyte line.

#### GLP general toxicological study in rats

A total of 142 rats (71/sex) were randomly assigned to 4 groups, i.e., Groups 1–4 with M102 dose levels by oral gavage at 0, 25, 50, and 75 mg/kg, respectively. The main study (toxicity study) animals were 10/sex/group, while Groups 1 and 4 had an additional 5/sex/group to assess recovery of any observed effects. Toxicokinetics (TK) animals were 3/sex in the control group and 6/sex/group in the treated groups. Animals were approximately 6–7 weeks of age with body weights ranging from 178.46 to 211.76 g in females and 252.21 to 295.14 g in males at dosing initiation. The dosage volume was 10 mL/kg. The control group (Group 1) was administered 10% (w/v) HP-β-CD in purified water (HP-β-CD) by oral gavage.

Criteria for evaluation included viability (morbidity/mortality), clinical observations, body weight, food consumption, ophthalmology, clinical pathology (hematology, coagulation, serum chemistry, and urinalysis), TK, gross pathology, organ weights, and histopathology. The concentration and homogeneity results of M102 in the dosing formulations met acceptance criteria, which demonstrated the formulations were accurately prepared and homogenous.

#### GLP general toxicological study in non-human primates (NHPs)

A total of 32 (16/sex) NHPs were randomly assigned to 4 groups including 5/sex/group in control and high dose groups, and 3/sex/group in low and middle dose groups. Dose groups were vehicle control [10% (w/v) HP-β-CD in purified water (HP-β-CD)] or M102 in vehicle at doses of 25, 50, or 75 mg/kg/day. At the end of the dosing phase, the last 2 surviving NHPs/sex/group in control and high dose groups were held for an additional 14 days without administration of the test article. Animals were approximately 2.4 to 2.9 months of age and with body weights ranging from 2.1 to 3.8 kg in males and 2.1 to 2.9 kg in females at dosing initiation.

Criteria for evaluation included viability (morbidity/mortality), clinical observations, body weight, food consumption, ophthalmic examinations, electrocardiograms, clinical pathology (hematology, serum chemistry, coagulation, urinalyses), gross (necropsy) evaluation, organ weight, histopathological evaluation and toxicokinetics.

#### Statistical analysis

Statistical analysis was performed using GraphPad Prism v11.00. Normality was assessed by the Shapiro-Wilk test. Unpaired t-test or the Mann Whitney test were used to compare 2 data points, depending on whether the samples presented a Gaussian distribution or not, respectively. Paired t-test was used to compare the same samples before and after treatment. One-way ANOVA followed by Dunnett’s multiple comparisons tests was used to compare more than 2 data points in 1 group. Two-way ANOVA followed by Šídák’s multiple comparisons tests was used to compare more than 2 data points in 2 groups.

## Discussion

Our understanding of the genetic underpinnings and pathophysiological mechanisms in ALS has substantially increased in recent years [9]. However, apart from the recent promising emergence of tofersen as a disease modifying therapy for the 2% of ALS patients who harbor mutations in the SOD1 gene, other approved drugs have only marginal effects on life expectancy (riluzole) or indices of disease progression (edaravone) [52]. Therapies targeting individual pathways have failed to generate significant benefit during several decades of clinical trials. Previous attempts at identifying effective neuroprotective therapies for ALS have failed, as they do not account for disease heterogeneity and the multiple mechanisms driving motor neuron injury [17, 53]. Data from disease model systems and from human biosamples provide strong evidence for a role of redox imbalance [40], inflammation [54], mitochondrial dysfunction [55] and altered proteostasis, including autophagy and mitophagy [56], as four key drivers in the pathobiology of ALS [9, 53]. Few, if any, studies in ALS have shown multi-target engagement for the proposed therapeutic agent in the clinic [17].

We previously identified M102, a CNS-penetrant, small molecule, activator of the NRF2-ARE pathway. Here, we have also shown that it also activates the HSF-1-HSE transcription factor pathway, which targets multiple genetically validated pathophysiological mechanisms in ALS. We identified M102 in a screen to discover CNS penetrant NRF2-ARE pathway activators [28]. M102 (S-apomorphine hydrochloride hemihydrate) is a proprietary new chemical entity (NCE) and the S-enantiomer of the marketed R-apomorphine (Apokyn^®^; pure R-enantiomer). The R-enantiomer is a dopamine agonist administered subcutaneously for the management of advanced Parkinson’s disease. M102 is a very weak dopamine antagonist and does not show the adverse effects associated with dopamine agonism [29]. Initial work showed beneficial effects in the *SOD1*^G93A^ mouse model and in indices of oxidative stress in ALS patient fibroblasts [28]. Here, we demonstrate that M102 is a dual activator of NRF2 and HSF1 transcription factor pathways, two upstream master regulators of neuroprotective mechanisms, with the potential to modulate all four of these key drivers of neurodegeneration and with excellent penetration across the blood brain barrier [34, 39].

NRF2 is a stress-responsive transcription factor and a master regulator of anti-oxidant and anti-inflammatory genes, activating the transcription of >1000 cytoprotective genes and with a multi-modal contribution to healthy mitochondrial function and regulation of key autophagy genes [57, 58]. These pleiotropic effects enable the cell to maintain homeostasis under stress conditions. NRF2 activity is tightly regulated via a complex set of transcriptional and post-translational controls, principally by KEAP1 in the cytoplasm and BTB domain and CNC homolog 1 (BACH1) in the nucleus [59]. Under physiological conditions, KEAP1 promotes constitutive proteasomal degradation of Nrf2 [60] while BACH1 acts as a transcriptional antagonist of NRF2 target genes [61]. Under oxidative stress conditions, KEAP1 is inactivated by modification of its reactive cysteine residues allowing NRF2 to escape degradation and newly synthesized NRF2 is able to translocate to the nucleus and bind to the anti-oxidant response elements (AREs) of multiple cytoprotective genes. This cytoprotective gene expression response provides protection against several key pathophysiological mechanisms operating in ALS including oxidative stress, mitochondrial dysfunction, inflammation and dysregulated proteostasis [18]. The stress response of the KEAP1-Nrf2-ARE system is stronger in astrocytes compared to neurons [43]. A body of evidence from in vitro and in vivo model systems and from post-mortem CNS tissue from ALS patients has indicated that the NRF2 response is impaired in ALS and has also been shown to decline with age [62–64].

HSF1 is a stress-inducible transcription factor that is the key driver for the expression of multiple heat shock proteins which act as chaperones responsible for the correct folding of newly synthesized proteins, the refolding of denatured proteins and the prevention of aggregation of misfolded proteins. This heat shock response is a pro-survival pathway activated under conditions of cellular stress. This cellular stress response declines with age, with decreased ability of HSF1 to bind to the promoters of genes encoding heat shock proteins [35]. Motor neurons have been reported to display a high threshold for the induction of the heat shock stress response [65]. Nevertheless, activation of the HSF1/HSP pathway has shown beneficial effects in cellular and animal models of ALS, including clearance of TDP-43 aggregates [66]. Activation of HSF1 is an attractive pharmacological target for ALS and other neurodegenerative conditions characterized by proteotoxic stress. However, to date, many small molecule activators of HSF-1 have shown undesirable properties e.g. by acting as Hsp90 inhibitors or by exerting direct proteotoxic effects [35, 67].

In vitro and in vivo data presented here show that M102 is a multi-target drug with predicted neuroprotective benefits for the treatment of ALS. As a dual activator of NRF2 and HSF1 pathways, M102 is able to activate anti-oxidative and anti-inflammatory pathways, as well as beneficial modulating effects on proteostasis and mitochondrial function. In vitro data also show that M102 can reduce TDP-43 proteinopathy and biomarkers of oxidative stress in patient-derived astrocytes. Moreover, M102 is capable of rescuing MN survival in co-cultures with iAstrocytes derived from *C9orf72*,* SOD1*, and sporadic ALS patients, as well as spinal cord MNs in the SOD1^G93A^ mouse model. The efficacy of M102 was shown in vivo in both *SOD1*^G93A^ and *TDP-43*^Q331K^ transgenic mouse models of ALS, with beneficial effects on a translatable neurophysiological biomarker (CMAP) which correlates with motor neuron survival in the spinal cord.

There are potential limitations to the in vivo models and study designs. The *TDP-43*^Q331K^ transgenic mouse model selected has low transgene expression and a relatively mild phenotype compared to the aggressive *SOD1*^G93A^ model. This is to avoid non-physiological TDP-43 overexpression. Phenotypes in this model do not significantly progress beyond 6 months of age and there is no reduction in survival [32]. This could be seen as a limitation of the study where a stronger phenotype with significant motor neuron loss could have been evaluated. For the *SOD1*^G93A^ mouse model we selected a relatively early timepoint for intervention. However, many behavioural, electrophysiological and neuropathological changes are occurring in mutant *SOD1* transgenic mice from the very earliest stages of development [50]. Our main objective in both models was to develop an exposure-effect relationship to enable prediction of efficacious doses in humans as we have been able to do. The selection of two different dosing regimes (once daily or twice daily, 5 mg/kg total dose in each case) in the *TDP-43*^Q331K^ model was based on the need to explore twice daily dosing which would give more options in terms of dosage from design in the clinical studies. However, the finding that oral M102 at 25 mg/kg was bioequivalent to SC dosed M102 at 5 mg/kg allowed us to revert to oral dosing for the *SOD1*^G93A^ study with the intent of pursuing once-daily oral dosage in clinical studies. In the *TDP-43*^Q331K^ model, some parameters show statistical significance in one dosing regime and not the other, however they are of the same or similar magnitude across dose groups for weight (AUC), gait analysis, CMAP and repetitive stimulation. We have interpreted the data in this context and consider that the different dosing regimes show broadly the same effect. The only exception to this is the rotarod data which show a transient statistical effect in the 5 mg/kg once daily group.

The finding that we observe a rescue of CMAP without a concomitant improvement in MN counts at 5 and 12.5 mg/kg in the *SOD1*^G93A^ mouse model is interesting and likely reflects the fact that axonal degeneration is occurring prior to complete loss of motor neuron cell bodies in the spinal cord as has been observed by others [68] and that lower doses of M102 do not sufficiently rescue this degenerative process.

The transcriptome of patient-derived astrocytes exposed in vitro to M102 confirm the activation of multiple key neuroprotective pathways in astrocyte lines from several subgroups of ALS. There is a degree of heterogeneity in the level of the in vitro response to M102, with the transcriptome of high responders shifting substantially towards the profile observed in the astrocytes of healthy controls.

This comprehensive package of pre-clinical efficacy data for therapy development in ALS is accompanied by strong safety data. We have completed the process of Good Manufacturing Practice (GMP) manufacturing for M102 and a series of GLP toxicology and safety pharmacology studies in rats and non-human primates, which have demonstrated that M102 will have an acceptable safety margin (9.3–15 for free drug) in clinical studies. Pharmacokinetic studies show strong penetration of M102 across the blood-brain-barrier, with a brain-plasma ratio of 0.7 at 30 min post-dosing.

Other NRF2 activators have been investigated in clinical trials or have been approved for medical use. These include dimethylfumarate (DMF) (Tecfidera^®^, Biogen) and omaveloxolone (Reata, Biogen). DMF was originally approved for the treatment of psoriasis (Fumaderm^®^) and was later repurposed for the treatment of relapsing-remitting multiple sclerosis (Tecfidera^®^). A phase 2 trial of DMF in ALS provided Class 1 evidence of safety at a dose of 480 mg/day and lack of disease-modifying efficacy [69]. DMF treatment is associated with dose-limiting lymphopenia and flushing (Tecfidera^®^ Prescribing Information). Omaveloxolone (Skyclarys^®^) is a potent NRF2 activator that has been approved by the FDA and EMA for the treatment of Friedreich’s ataxia. By activating the NRF2 pathway, omaveloxolone ameliorates oxidative stress and improves mitochondrial function. As a potent NRF2 activator, omaveloxolone exhibited significant liver toxicity with elevated AST/ALT levels in 37% of patients exposed to a dose of 150 mg [70]. Toxicity has also been reported with other potent NRF2 activators, such as bardoxolone methyl (EC50: 53 nM) which showed significant heart, liver, and renal toxicity in humans [71]. In contrast, our preclinical toxicological studies indicate that M102 has a much higher safety margin in relation to liver toxicity. Arimoclomol is a co-activator of HSF1 and has been ineffective in two clinical trials targeting *SOD1*-ALS initially and then ALS more broadly [72, 73].

There is a huge unmet need for more effective neuroprotective therapies to slow disease progression in ALS. Here, we have demonstrated that M102, a combined activator of NRF2 and HSF1 signalling pathways, has positive therapeutic effects in two different ALS transgenic mouse models and improves motor neuron survival and multiple pathological markers (i.e. oxidative stress, misfolded SOD1, TDP-43 proteinopathy) in a range of human ALS cellular model systems. Importantly, these neuroprotective effects are seen across multiple subtypes of ALS, including the two most common genetic subtypes caused by mutations in the *C9orf72* and *SOD1* genes, as well as sporadic ALS cases. Taken together, M102 has the potential to modulate multiple key drivers of neurodegeneration, increasing the chances of achieving impactful neuroprotection and disease modifying effects in ALS. Its positive effect on TDP-43 proteinopathy and on rescuing motor neuron survival both in vivo and in co-culture with iAstrocytes derived from both familial and sporadic ALS patients also suggest that M102 may be beneficial for a wide range of ALS subtypes as ~ 97% of ALS cases display TDP-43 proteinopathy and ~ 90% of cases are sporadic.

## Supplementary Information

Below is the link to the electronic supplementary material.

Supplementary Material 1

## Data Availability

Materials used in this study can be made available subject to Materials Transfer agreements (MTAs). RNA Sequencing data will be made available on an appropriate public database following publication of the manuscript. All other data are available in the manuscript main text and supplementary materials.

## Declarations

### Patents relevant to M102

PCT/US2019/056996 - Treatment of Neurodegenerative diseases. Inventors: Ning Shan, Richard Mead, Laura Ferraiuolo, Pamela J Shaw. This covers the mechanism of action of a drug identified at SITraN for the treatment of neurodegenerative diseases, including ALS. Submitted in 2019. PCT/US2019/056998 - Treatment of Neurodegenerative diseases. Inventors: Laura Ferraiuolo, Ning Shan, Pamela J Shaw. This covers the specific properties of a neuroprotective compound identified at SITraN. Submitted in 2019. PCT/US2020/45321 - Pharmaceutical Composition For Use In The Treatment Of Neurological Diseases. Inventor: Ning Shan. This covers the pharmaceutical compositions and associated pharmacokinetics of a drug identified at SITraN. Submitted in 2019.

## Abbreviations

ALS: Amyotrophic lateral sclerosis
ARE: Anti-oxidant response element
ASO: Anti-sense oligonucleotide
AUC_last_: Area under the plasma concentration-time curve to the last measurable plasma concentration
BACH1: BTB domain and CNC homolog 1
BCA: Bicinchoninic acid
C9orf72: Chromosome 9 open reading frame 72
CMAP: Compound muscle action potential
CMAX: Maximum plasma concentration
CNS: Central nervous system
CSF: Cerebrospinal fluid
DEG: Differentially expressed gene
DMF: Dimethylfumarate
DTT: Dithiothreitol
ELISA: Enzyme -linked immunosorbent assay
EMA: European Medicines Agency
FDA: Food and Drug Administration
FFPE: Formalin-fixed, paraffin-embedded
FTD: Fronto-temporal dementia
GAPDH: Glyceraldehyde-3-phosphate dehydrogenase
GLP: Good Laboratory Practice
GMP: Good Manufacturing Practice
GRASPS: Genome-wide RNA analysis of stalled protein synthesis
GSH: Glutathione
HSF1: Heat shock factor 1
HSE: Heat-shock element
HSP70: Heat shock protein 70
IACUC: Institutional Animal Care and Use Committee
ICC: Immunocytochemistry
IHC: Immunohistochemistry
iNPC: Induced neural progenitor cell
KEAP1: Kelch-like ECH-associated protein 1
M102: S-(+)-10,11-dihydroxyaporphine
NCE: New chemical entity
NFR2-ARE: NF-E2 p45-related factor 2:antioxidant response element
NHP: Non-human primate
NMJ: Neuromuscular junction
NOAEL: No observed adverse effect level
NQ01: NAD(P)H dehydrogenase [quinone]1
8-OHG: 8-oxo-2-oxyguanosine
PCA: Principal component analysis
PK: Pharmacokinetic
sALS: Sporadic amyotrophic lateral sclerosis
SOD1: Cu-Zn superoxide dismutase 1
TDP-43: Transactive response DNA binding protein 43
TK: Toxicokinetics
WT: Wild type
