# Supplementary material for: M102 activates both NRF2 and HSF1 transcription factor pathways and is neuroprotective in cell and animal models of amyotrophic lateral sclerosis
Source: Mol Neurodegener. 2025 Nov 4;20:118. doi: 10.1186/s13024-025-00908-y (PMC12584299; doi:10.1186/s13024-025-00908-y)
Supplement: Supplementary file 1 — Supplementary Material 1 [file 13024_2025_908_MOESM1_ESM.docx]

**SUPPLEMENTARY MATERIAL**

**Title: M102 activates both NRF2 and HSF-1 transcription factor pathways and is neuroprotective in cell and animal models of amyotrophic lateral sclerosis.**

Authors: Amy F. Keerie^1,2†^, Raquel Rua Martins^1,2†^, Chloe F. Allen^1,2†^, Katie Bowden^1,2^, Sufana Al Mashhadi^1,2^, Thomas Marlow^1,2^, Monika Myszczynska^1,2^, Nikitha Thakur^1,2^, Selina N. Beal^1,2^, Allan Shaw^1,2^, Shivani Suresh^1,2^, Scott N. McKinnon^1,2^, Johnathan Cooper-Knock^1,2,4^, Ryan J.H.West^1,2^, Sam Bonsall^1,2^, Alex Daniel^1,2^, Tyler Wells^1,2^, V Kumar^1^, Brittany C.S. Ellis^1^, Maureen Higgins^5^, Albena T. Dinkova-Kostova^5^, Tatyana A. Shelkovnikova^1,2^ , Ira N. Kalfus^3^, Ning Shan^3^, Pamela J. Shaw^1,2,4^‡*, Laura Ferraiuolo^1,2^‡, Richard J. Mead^1,2^‡

**SUPPLEMENTARY MATERIALS AND METHODS: *IN VIVO***

**Pharmacokinetic study in rodents**

Pharmacokinetics of M102 was evaluated via single-dose subcutaneous and oral administrations of M102 in male C57Bl/6 mice. For subcutaneous administration, nine male C57BL/6J mice were administered with a single dose of 5 mg/kg M102. Blood samples were collected from each animal at 0.0833, 0.167, 0.25, 0.5, 1, and 2 hr post dose. For oral administration, a total of 18 male C57Bl/6 mice were dosed at 10 mg/kg, with three animals per blood sampling time point, which was determined as 0.25, 0.5, 1, 2, 3, and 6 hours post dose. Following blood collection from individual animals, plasma samples were extracted and processed, and then analysed by an LC-MS/MS bioanalytical method to determine the M102 plasma concentrations.

To investigate the brain exposure of M102, additional pharmacokinetic studies were also conducted in Spray-Dawley rats. Single dose oral administration of M102 at 12 mg/kg was given to two groups of male rats (N= 3/group). Plasma and brain samples were collected at 30 or 60 min post dose for two individual groups. Bioanalyses were conducted for plasma and brain samples to determine the M102 concentrations via LC-MS/MS.

Data acquisition was performed by LabSolutions version 5.89 Software (Shimadzu). All concentration data were reported with 3 significant figures. Data statistical analysis was performed using Microsoft Excel® software. The PK parameters of M102 were calculated using a non-compartmental approach with Pharsight® WinNonlin® v5.2 software package.

**General toxicology study in rats and non-human primates (NHPs)**

GLP general toxicology studies in rats and NHPs were conducted at WuXi AppTec (Suzhou, China) to evaluate potential toxicity of M102 when administered once daily for 28 days by oral gavage and to assess the reversibility, persistence, or delayed occurrence of toxic effects following a 14-day recovery period.

M102 at doses of 25, 50 or 75 mg/kg/day by oral gavage was investigated in the study of each species, based on previous non-GLP general toxicology study results. Criteria for evaluation included: viability (morbidity/mortality), clinical observations, body weight, food consumption, ophthalmology, clinical pathology (hematology, coagulation, serum chemistry, and urinalysis), toxicokinetics (TK), gross pathology, organ weights, and histopathology. Based on the study outcomes, no observed adverse effect levels (NOAELs) in either rats or NHPs were determined.

The protocol and additional amendments involving the care or use of animals involved in this study have been reviewed and approved by WuXi AppTec Institutional Animal Care and Use Committee (IACUC) prior to the initiation of study procedures. In addition, an onsite staff veterinarian monitored the study for animal welfare issues.

Toxicokinetic (TK) analysis of M102 plasma concentration-time data was performed at the Testing Facility. TK parameter values, including the maximum plasma concentrations (Cmax), the time to reach the maximum concentrations (Tmax), and the area under the plasma concentration vs. time curve (AUC) from time zero to 24 hours post-dose (AUC0-24h), were determined using a validated WinNonlin program. Values that were below the lower limit of quantification (BLQ) were set to zero in the calculation of the mean concentrations. Male and female TK data were analyzed separately. An audited TK report was generated and is attached to the Final Report as an appendix. Safety margin calculations were performed based on the resulting TK data and available preclinical *in vivo* PK data in mice, using the WinNonlin program.

**Murine therapeutic study design**

Transgenic female mice were block randomised into the different dose groups. Time points for behavioral tests were determined through analysis of previous characterisation of the models. The number of mice per group was determined through power analysis based on the standard deviation seen in the read-outs in previous experiments. Power analysis was based on our previously published model characterisations (32, 50).

**Dosing**

The oral formulation of M102 was in 10% HP-b-CD in water, which was filter sterilised before addition of M102. The subcutaneous formulation of M102 was in 1% ascorbic acid (Sigma-Aldrich), 0.05% sodium metabisulfite (Sigma-Aldrich) and 0.9% saline adjusted to pH 3.5. This was filter sterilised before the addition of M102. M102 was weighed out into bijous and protected from light. On the morning of dosing M102 was dissolved in the appropriate vehicle for either oral or subcutaneous dosing to create the correct concentrations. Formulation of M102 was aided by vortexing and heating. Mice were dosed at 10 ml/kg and body weight was recorded daily for calculating doses.

**Rotarod**

Animals were tested once per week (TDP-43^Q331K^ mice) or twice per week (SOD1^G93A^ mice) on an accelerated rotarod (ACCELER rota-rod for mice, Jones & Roberts). The rotarod protocol used accelerated from 4 to 40 rpm over 5 minutes. Before testing started, mice were acclimatised to the equipment for three consecutive days with two runs per day. On test days, the mice were run twice on the rotarod protocol with a rest period in between and the time taken to fall off the equipment was recorded. Analysis was carried out using the best performance run on each testing day.

**Catwalk gait analysis**

Gait analysis was conducted using the Catwalk gait analysis system (Noldus), which consists of a glass plate and a green LED inside that is internally reflected when paws make contact with the surface and a camera mounted underneath. Mice ran backwards and forwards on the glass until 6 straight continuous runs were collected. Analysis was carried out with Catwalk software using 3 runs per mouse (Noldus version 7.1) with further analysis carried out using excel (Microsoft) and Graphpad prism.

**Electrophysiology: compound muscle action potential (CMAP) and repetitive stimulation**

Mice were anesthetised with 5% isoflurane and 4L/min oxygen in an induction box and then maintained with a nose cone under 2% isoflurane and 0.5L/min oxygen, which was adjusted as needed per mouse to keep breathing regular. The left hind limb of the mouse was then shaved using an electric razor and the fine fur was removed using hair removal cream (Veet). After loss of pedal reflex, a grounding electrode (Ambu Neuroline) was placed in the tail of the mouse, with the reference ring electrode being placed around the ankle covered in Ten20 nerve conductive paste (Weaver and Company) to ensure good contact with the skin. The recording ring electrode was then placed around the top part of the leg. The sciatic nerve was stimulated with a square input pulse with a 0.1ms duration, using a pair of twisted electrodes (Ambu Neuroline) that stimulated the hind limb. The CMAP amplitude was detected by the reporting electrode for the hind limb and the amplitude was recorded. The starting stimulating amplitude was approximately 5mV which was then increased gradually until the supramaximal response was determined for the CMAP, which was recorded for analysis. Analysis was carried out using Graphpad prism.

Repetitive stimulation was carried out by stimulating the muscles at the supramaximal amplitude for 10 stimuli at 10Hz to determine if the response declined over time. The amplitude of each response was recorded and analysis was carried out using excel to determine percentage change in stimulations between 1st and 10th response. Further analysis was carried out using Graphpad prism.

**Tissue collection**

At the end of the studies, mouse tissues were collected for histological analysis through perfusion or for protein and RNA analysis through a method of snap freezing and exposure to RNA later. Mice for perfusion were overdosed with pentobarbital and, after loss of the pedal reflex, were perfused with PBS followed by 4% paraformaldehyde. Brain and spinal cord were removed and stored in 4% PFA overnight before being stored in PBS.

**Preparation of lumbar spinal cord sections and histology**

A 12mm segment of lumbar enlargement was collected from the PFA fixed animals and embedded in two halves in paraffin wax. Sections were cut at a thickness of 10 μm and placed onto glass slides in a serial manner in blocks of 5 slides. Paraffin was removed from the sections, and they were rehydrated by washing in 100% xylene and a decreasing concentration gradient of ethanol.

**Nissl staining**

Slides were incubated with 0.1% cresyl violet for 15 minutes and then washed in water and incubated in acetic acid for 4 – 8 seconds followed by 100% ethanol for 1 minute and 100% xylene for 1 minute. These were then mounted and imaged using a nanozoomer digital slide scanner (Hamamatsu). Motor neuron counting was performed blinded and the criteria for counting of motor neurons were: cell body size greater than 20 μM in any axis excluding processes; location in the spinal cord ventral horn; and the presence of a nucleus and nucleolus.

**RNA extraction**

RNA was extracted from brain and spinal cord using the RNeasy lipid mini kit (Qiagen, 74804) following the manufacturer’s protocol. RNA concentration and purity were checked using a nanodrop ND-1000 spectrophotometer (Thermo Scientific) by measuring absorbance at 230, 260 and 280 nm.

**cDNA synthesis**

cDNA was synthesised from RNA by firstly digesting DNA from 2000ng of RNA using RNase-free DNase and DNase buffer (Roch Diagnostics). Random hexamer primers and deoxyribonucleotide triphosphates were added to each reaction and incubated at 75°C for 5 minutes to denature RNA, and samples were placed immediately on ice. Reverse transcriptase was added (Invitrogen, 28025-013) to all samples and these were placed in a PCR machine with the following protocol: 25°C for 10 minutes, 42°C for 1 hour, 85°C for 5 minutes.

**RT-qPCR**

RT-qPCR was carried out on cortex and spinal cord cDNA using optimised primers for targets that were downstream of NRF2 or HSF1. Gapdh was used as an endogenous control and data were analysed using the delta-delta method to gain relative gene expression to vehicle dosed samples.

**MATERIALS AND METHODS: *IN VITRO***

**Evaluation of HSE-luciferase inducer activity**

HeLa-HSE-luciferase cells express luciferase under the transcriptional control of the human Hsp70.1 promoter (75). Cells were seeded (1 × 104 cells/well) into white flat-bottomed 96-well plates (Thermo Scientific). On the following day, the cells were treated in 8 replicates with a range of concentrations of compounds (e.g. 1.25, 2.5, 5, 10, 20, 40 µM), or their respective vehicle 0.1% (v/v) DMSO]. The final solvent concentration in the cell culture medium was maintained at 0.1% (v/v) in all wells. After 24 hours, 50 µl of Bright-Glo luciferase assay reagent (Promega) was added directly into the medium in each well, followed by gentle shaking of the plates at room temperature for 5 minutes. The luminescence was measured (5-second read per well) in half of the wells treated by each compound using a microplate reader. The media was removed from the other half of the plates. The cells were then washed twice with PBS, followed by addition of 50 µl of lysis buffer [50 mM Tris-HCl pH 7.5, 10 mM KCl, 5 mM MgCl2, 0.5% Nonidet P40, 1 mM dithiothreitol (DTT)] into each well. Twenty microlitres of the resulting lysate from each well was used to perform bicinchoninic acid (BCA) protein assay in order to determine the protein concentration of the lysate and normalize the obtained luminescence values.

**Evaluation of the requirement for NRF2 and HSF1 for induction of NQO1 and Hsp70**

SHSY5Y cells were seeded (2.5 × 105 cells/well) into 6-well plates in DMEM/F12(1:1), from Gibco, supplemented with 10% heat-inactivated FBS. Cells were diluted in complete growth medium without antibiotics so that 1.5 ml contained 2.5 × 105 cells. 1.5 ml of cell suspension was pipetted into each well. A 5 µl volume of Lipofectamine RNAiMAX was pipetted into each tube containing 500 µl Opti-MEM Medium without serum in a 50 ml tube. The appropriate amount of RNAi duplex was added to the tube, mixed gently and incubated for 10-20 minutes at room temperature. A 500 µl volume of RNAi duplex-Lipofectamine RNAiMAX complexes was added to each of the wells containing cell suspension. This gives a final volume of 2 ml. The wells were mixed gently by rocking the plate back and forth. The cells were grown for a further 64 hours at 37°C in a 5% CO2 incubator.

After this, the cells were treated with M102 at concentrations indicated in the figures, in the presence or the absence of Cu2+ (50 µM) for 16 hours. At the end of treatment, the cells were lysed in 1X SDS Lysis Buffer (50 mM Tris/HCL pH 6.8, 2% SDS (w/v), 10% Glycerol (v/v), 0.02% Bromophenol blue(w/v)), and the whole cell lysates were transferred to a 1.5 ml tube, sonicated for 4x5 secs at 20%.

The proteins from the cell lysates were separated by SDS-PAGE on a 4-12% MOPS gel, then transferred to nitrocellulose membranes for western blotting. They were then probed for Hsp70, Nqo1 and GAPDH.

**Immunohistochemistry to demonstrate a marker of oxidative stress in human postmortem CNS tissue**

Formalin-fixed, paraffin-embedded (FFPE) human CNS post-mortem tissue obtained from the Sheffield Brain Tissue Bank was used to investigate the level of nucleic acid oxidation in ALS patients versus controls using 8-OHdG as a marker of oxidative stress. Using a case control structure, the cohort consisted of ALS samples with age matched controls of: (i) frontal cortex (Fcx); (ii) motor cortex (Mcx) and (iii) spinal cord (SC). Immunohistochemistry was carried out on sections from 30 FFPE blocks derived from 10 individuals: 3 controls, 6 sALS, 2 C9-ALS and 2 C9 ALS-FTD (**Supplementary Table 4**).

FFPE blocks were sectioned at 6 μm and immunohistochemistry was performed using a standard ABC method (Vector Laboratories, Peterborough, UK) and the signal visualized using 3,3′-diaminobenzidine. A summary of the primary antibody and the conditions, including antigen retrieval, is shown in **Supplementary Table 9**. Negative controls consisted of sections incubated with isotype controls or with omission of the primary antibody.

**Detection of oxidative stress in human CSF by enzyme linked immunosorbent assay (ELISA)**

The level of expelled fractions of oxidised material in the CSF of ALS patients and controls was determined by ELISA to detect the oxidised RNA fraction. RNA oxidative stress has been reported to be directly associated with neurodegenerative pathology. The level of RNA oxidation was measured in 25 human CSF samples: 13 ALS patients and 12 controls (**Supplementary Table 5**) using the Cell Bio-labs Oxiselect RNA 8-OHdG ELISA kit (Catalog Number STA- 325). The unknown 8-OHG CSF samples or 8-OHdG standards were first added in absolute concentration to an 8-OHdG/BSA conjugate preabsorbed EIA plate. Following a short incubation, an anti-8-OHdG monoclonal antibody was added, followed by an HRP conjugated secondary antibody. The quantity of 8-OHdG in the unknown sample was determined by comparing its absorbance with that of a known 8-OHdG standard curve. The kit has an 8-OHdG detection sensitivity range of 300 pg/mL to 40 ng/mL.

**Immunofluorescence imaging of iAstrocytes.**

After fixation in 4% PFA for 10 min at RT, cells were washed (2x 5 min) in PBS (137 mM NaCl, 2.68 mM KCl, 10.14 mM Na2HPO4, 1.76 mM KH2PO4), blocked and permeabilised in 5% horse serum (DAKO) with 0.5% triton X-100 (AppliChem) for 30-60 min at RT, and incubated in primary antibody ON at 4^o^C (details of the primary antibodies in **Supplementary Table 10**). The cells were then washed (1x 5min in 0.2% Tween-20, Sigma, and 2 x 5min in PBS) and incubated in 1:1000 dilution of secondary antibody for 30-60min at RT (details of the secondary antibodies in **Suppl. Table 10**). Finally, the cells were stained with Hoechst 33342 (1:6000, Life Technologies) for 5-10 min at RT before washing (as previously) and then stored at 4°C. Imaging was performed with the OPERA Phoenix high-content imaging system (Perkin Elmer). Harmony 4.9 Analysis Software (Perkin Elmer) was used to analyse and quantify the intensity of immunostaining.

**Immunoblotting of iAstrocyte lysates**

Protein extraction was performed by lysing the cells in IP lysis buffer (150 mM NaCl, 50 mM HEPES, 1 mM EDTA, 1 mM DTT, 0.5% (v/v) Triton X-100, PIC, pH 8.0) containing protease and phosphatase inhibitors, for 15 min on ice, followed by a centrifugation at 17,000g for 5 min at 4°C. Alternatively, the cells were lysed with RIPA buffer with 10% proteinase inhibitor and sonicated using the Soniprep 150 (MSE) for 10 seconds at 25% amp. before centrifuging as previously. The supernatant was removed and discarded, and this last step was repeated to wash the cell pellet, ensuring that all RIPA-soluble protein had been removed before the addition of urea. The supernatant was replaced with 10 μl of urea buffer and the sample was pipetted a few times to solubilise RIPA-insoluble material. The protein lysates were quantified using a bicinchoninic acid (BCA) assay (BCA assay kit, ThermoFisher Scientific), following manufacturer’s instructions, or by Bradford assay.

A total of 20-30 µg of protein mixed with 4x Laemmli buffer (228 mM Tris-HCl, 38% (v/v) glycerol, 277 mM SDS, 0.038% (v/v) bromophenol blue, 5% (w/v) β-mercaptoethanol pH 6.8) was denaturated at 95°C for 5 min and loaded into an SDS-polyacrylamide gel (12% resolving gel and 5% stacking gel). Resolving gel: 3.5 ml of dH2O2, 1.5 ml of 30% acrylamide, 2.5 ml of resolving buffer (1.5M Trizma®, 13.9mM SDS, pH 8.8, filtered), 50 µl of 10% APS, 20 µl of TEMED. Stacking gel: 5.8 ml of dH2O2, 1.7 ml of 30% acrylamide gel, 2.5 ml of stacking gel (0.5M Trizma®, 13.9 mM SDS, pH 6.8, filtered), 50 µl of 10% APS, 20 µl of TEMED. The SDS-polyacrylamide gel, assembled into a Mini-PROTEAN® Tetra Vertical Electrophoresis Cell (BioRad) filled with running buffer (25 mM Tris, 3.5 mM SDS, 20 mM glycine), was run for approximately 1.5h at 150-180V using a PowerPac Basic (BioRad). Then, the protein was transferred from the gel onto a nitrocellulose membrane (GE Healthcare) pre-soaked in transfer buffer (47.9 mM Tris, 38.6 mM glycine, 1.38 mM SDS, 20% (v/v) methanol), using the semi-dry Biometra FastblotTM transfer system (Analytik Jena), at 0.15A/gel for 1h.

The nitrocellulose membrane was blocked for 1h in 5% (w/v) milk in tris-buffered saline with Tween-20 (TBST) (20 mM Tris, 137 mM NaCl, 0.2% (v/v) Tween® 20, pH 7.6), at RT. After blocking, the membranes were incubated in primary antibody (**Supplementary Table 11**) diluted in blocking buffer, ON at 4°C. After 3x 5 min washes in TBST, the membrane was incubated in secondary antibody (anti-rabbit IgG HRP, or anti-mouse IgG HRP, Promega) diluted in blocking buffer for 1 h at RT. The membrane was then washed as previously, incubated in ECL for 1 min and scanned using the Odyssey® Fc Imaging System from LI-COR Biosciences.

**Genome-wide RNA analysis of stalled protein synthesis (GRASPS) to detect translating mRNAs.** (76)

**RNA extraction and ribosomal purification:**

RNA was extracted in Buffer A (250 mM sucrose, 5 mM KCl, 50 mM Tris-HC) containing proteinase inhibitor, 2 mM PMSF and 0.16 U/μl RNAse inhibitor. A final concentration of 0.7% v:v NP-40 from a 10% stock solution was gently mixed into the lysate. The lysate was incubated on ice for 5 minutes, mixed and incubated on ice for another 5-10 minutes. On a 6 well plate, the lysate was UV-irradiated on ice at 0.3 J/cm² and then centrifuged at 750 g for 10 minutes at 4°C to pellet the nuclear fraction. This nuclear pellet was discarded, and the supernatant was centrifuged at 12,500g for 10 minutes at 4°C to pellet the mitochondrial fraction. The supernatant, (post-mitochondrial (PMT) fraction) was transferred to a new cold Eppendorf tube and 4 M KCl was added to give a final concentration of 0.5M KCl. 1 ml of sucrose cushion was added to the bottom of clean, cold TLA100 centrifuge tubes. The PMT fraction was made up to 1 ml with Buffer B (250 mM sucrose, 500 mM KCl, 50 mM Tris-HCl, 5 mM MgCl2) and 900 μl of the 0.5 KCl-adjusted PMT fraction was slowly dispensed on top of the sucrose cushion and the tubes were balanced precisely within 0.01 g of each other before centrifuging at 250,000 g/75,000 rpm for 2 hours at 4°C in the TL-100 benchtop ultracentrifuge (Beckman). The ribosome pellet was quickly washed with cold DEPC water before resuspending in 250 μl of ribosome resuspension buffer (RRB). 0.16 U/μl Ribosafe RNAse inhibitor and 100 μg/ml proteinase K were added to the RRB and this was incubated for 30 minutes at 37°C with a pulse vortex every 5 minutes. After incubation, 10 mM EDTA and 50 mM NaAc was added and the RRB was vortexed. 750 μl of PureZOL RNA isolation reagent was added and left for 10 minutes at room temperature before the RNA was extracted using the Direct Zol RNA Miniprep Plus kit.

**mRNA purification**: The mRNA was isolated using the NEB Next® Poly(A)+ mRNA Magnetic Isolation Module. 15 μl of Oligo d(T)25 beads were dispensed into 0.2 ml tubes and the beads were washed twice with 100 μl of 2 x RNA binding buffer to ensure removal of the supernatant. An equal volume (50 μl) of 2 x RNA binding buffer and sample RNA were added to the beads. The samples were incubated at 65°C for 5 minutes and immediately placed on ice for 2 minutes. The samples were incubated at room temperature for 5 minutes before they were placed on a DynaMagTM-2 magnet (Life Technologies) for 2 minutes. The supernatant was removed and kept on ice. Previous work investigating this technology had incorporated an additional binding step which increased the yield of ribosomal poly(A)+ RNA. The beads were washed twice with 200 μl of wash buffer and placed on the magnetic rack for 2 minutes. The beads were then stored on ice while the initial saved supernatant was heated to 65°C for 5 minutes and directly on ice for 2 minutes. The supernatant was then re-added to the beads and the binding of the poly(A)+ RNA was repeated as above. After the supernatant was removed and kept on ice, the beads were washed twice with 200 μl wash buffer. After ensuring the total removal of the wash buffer, 50 μl of Tris-buffer was added to the beads. The samples were incubated at 80°C for 2 minutes then immediately left at room temperature. 50 μl of RNA binding buffer was then added to the beads and the samples were incubated at room temperature and inverted every few minutes before placing the tubes on the magnetic rack and removing the supernatant. The beads were washed twice again in 200 μl of wash buffer. The poly(A)+ mRNA was eluted from the beads by adding 20 μl of Tris-buffer, incubating samples at 80°C for 2 minutes and then placing on the magnetic rack to transfer the supernatant to a new nuclease-free PCR tube. The sample was then added to the beads and the binding of the poly(A)+ was repeated as described above. After this incubation, the tubes were placed on the magnetic rack to remove the supernatant. The mRNA was again eluted from the beads through the addition of 20 μl of Tris-buffer and the process described above. The quantity of purified mRNA was assessed using the NanoDrop Spectrophotometer ND-1000 (Labtech International).

**Ribosomal RNA depletion**: 3 μl of the RNA/probe Master Mix (1 μl of NEBNext rRNA depletion solution, 2 μl probe hybridisation buffer) was added to 12 μl of RNA sample and pipetted up and down 10 times. The samples were briefly spun in a tabletop centrifuge and immediately placed in the G-STORM thermocycler (LABCARE) with a heated lid of 80°C running the following program: 95°C for 2 min, 22°C for 5 min. The samples were spun down briefly and placed on ice while the RNase H Digestion Master Mix (2 μl NEBNext RNase H, 2 μl of RNase H reaction buffer, 1 μl nuclease-free water). 5 μl of the above mix was added to each sample and was mixed thoroughly with a pipette. The samples were briefly spun and immediately placed in a thermocycler with a heated lid of 40°C and incubated at 37°C for 30 minutes. After incubation, the samples were spun down briefly again and placed on ice while the DNase I Digestion Master Mix (5 μl DNAse I reaction buffer, 2.5 μl DNase I, 22.5 μl nuclease-free water). 30 μl of the above mix was added to each sample and thoroughly mixed with a pipette. The samples were briefly spun and immediately incubated at 37°C for 30 minutes in the thermocycler. After incubation, the samples were spun down again and placed on ice. A 1 ml aliquot of NEBNext® RNA Sample Purification Beads was vortexed into suspension and 110 μl of this suspension was added to each sample. The samples were then thoroughly mixed with a pipette and incubated on ice for 15 minutes. The tubes were placed on a magnetic rack to separate the beads from the supernatant. After 5 minutes, the supernatant was removed and discarded, and the beads were washed twice with 200 μl of freshly prepared 80% ethanol; beads were incubated in ethanol for 30 seconds between washes. The tubes were briefly spun to remove any excess ethanol. The beads were then air dried for 5 minutes while the tubes were left on the magnetic rack with the lids open. The samples were eluted while the beads were still dark brown and glossy looking; if the beads were over-dried this would impact the yield. 8 μl of nuclease free water was added to the beads, thoroughly mixed, and incubated on ice for 2 minutes to elute the RNA sample from the beads. The tubes were placed on the magnetic rack for 5 minutes to sediment the beads and the RNA sample was transferred to a fresh 0.2 μl Eppendorf.

**RNA Sequencing**

RNA sequencing was performed at the Centre of Genomic Research, University of Liverpool, using a dual-indexed, strand-specific RNA-Seq library from the submitted poly(A)+ enriched RNA sample using the NEB Next Ultra Directional RNA library preparation kit. Samples were sequenced on lanes of the Illumina Nova Seq using S4 chemistry (paired-end, 2 x 150 base pair sequencing) which generated approximately 2500 M clusters per lane. RNA sequencing data were quality controlled at the centre by removal of any low-quality bases and sequencing adapters.

**Quality control:** Bcbio is a python toolkit providing best-practice pipelines for fully automated high throughput sequencing analysis (ttps://bcbio-nextgen.readthedocs.io/en/latest/). The bcbio pipeline was used to merge all 5-6 FastQ files per sample together for quality control and further processing. Inspecting the GC-content histograms from FastQC https://www.bioinformatics.babraham.ac.uk/

projects/fastqc/) revealed a bimodal distribution for some samples attributed to ribosomal contamination. Therefore, the bbsplit tool was used to remove any residual rRNA sequences prior to alignment.

**Sequence alignment and differential expression**: Salmon (https://salmon.readthedocs.io/en/latest/) was used to quantify the expression of transcripts using the RNA sequencing data and to perform an inference step to estimate the relative abundance of all the known transcripts without aligning the reads. The genecode set of transcripts (v28) was used for quantification, giving a matrix of transcript-level quantifications for each sample. The tximport Bioconductor package was used to aggregate the counts to the gene-level and these counts were imported into the DESeq2 Bioconductor package for quality control and analysis. Visualisation of the raw count distributions and unsupervised analysis prompted the removal of two outlier subjects from further analysis (control 155 and C9orf72 patient 183). The DESeq2 differential expression method incorporates gene-length and library size correction as part of the model. Prior to visualisation in other analysis tools, the RNA sequencing data were normalised by the FPKM (fragments per kilobase per million reads) method. Lists of genes that were statistically differentially expressed were identified by applying a p-value <0.05 and absolute log2 fold-change >1.5. The gene lists were inputted into DAVID Functional Annotation Bioinformatics Microarray Analysis programme (https://david.ncifcrf.gov/). The list of Gene Ontology Term “Biological Process” (GO-BP) terms were exported from DAVID to categorise differentially expressed genes (DEG). To visualise group segregation based on DEGs, principal component analysis (PCA) plots and heatmaps were generated using Qlucore Omics Explorer software (Qlucore).

**Assessment of TDP-43 regulated cryptic splicing in sALS i-Astrocytes**

Cryptic splicing in the two genes with high astrocytic expression, ATG4B and EXD3, in control and sALS iAstrocytes, was analysed by RT-qPCR. Then we assessed the level of expression of these genes following exposure to M102 for 48-96 hours. RNA samples were prepared from DMSO- or M102-treated iAstrocytes as described above, and utilised for first-strand cDNA synthesis, which was performed with 500 ng of RNA per sample using random primers (ThermoFisher) and MMLV reverse transcriptase (Promega), as per manufacturer's protocol. qRT-PCR was performed using qPCRBIO SyGreen Lo-ROX (PCRbio), and RPL13A was used for normalisation (housekeeping gene). Primers were designed based on the cryptic exon coordinates as in (48). Primers for cryptic splicing analysis were validated previously (49): EXD3, 5’-CTCCAGTGAGACAACCCTG-3’ and 5’-GCAAGCTGAAAGGTCTGTGA-3’; ATG4B, 5’-TCATAACCTGCCCCAATCCC-3’ and 5’-CCTTGATTCGCCCACGAGAT-3; RPL13A 5’-CTGGTCCTGGGTTGGAGTTG-3’ and 5’-CAAGCGGATGAACACCAACC-3’.

**Supplementary Figures and Tables**


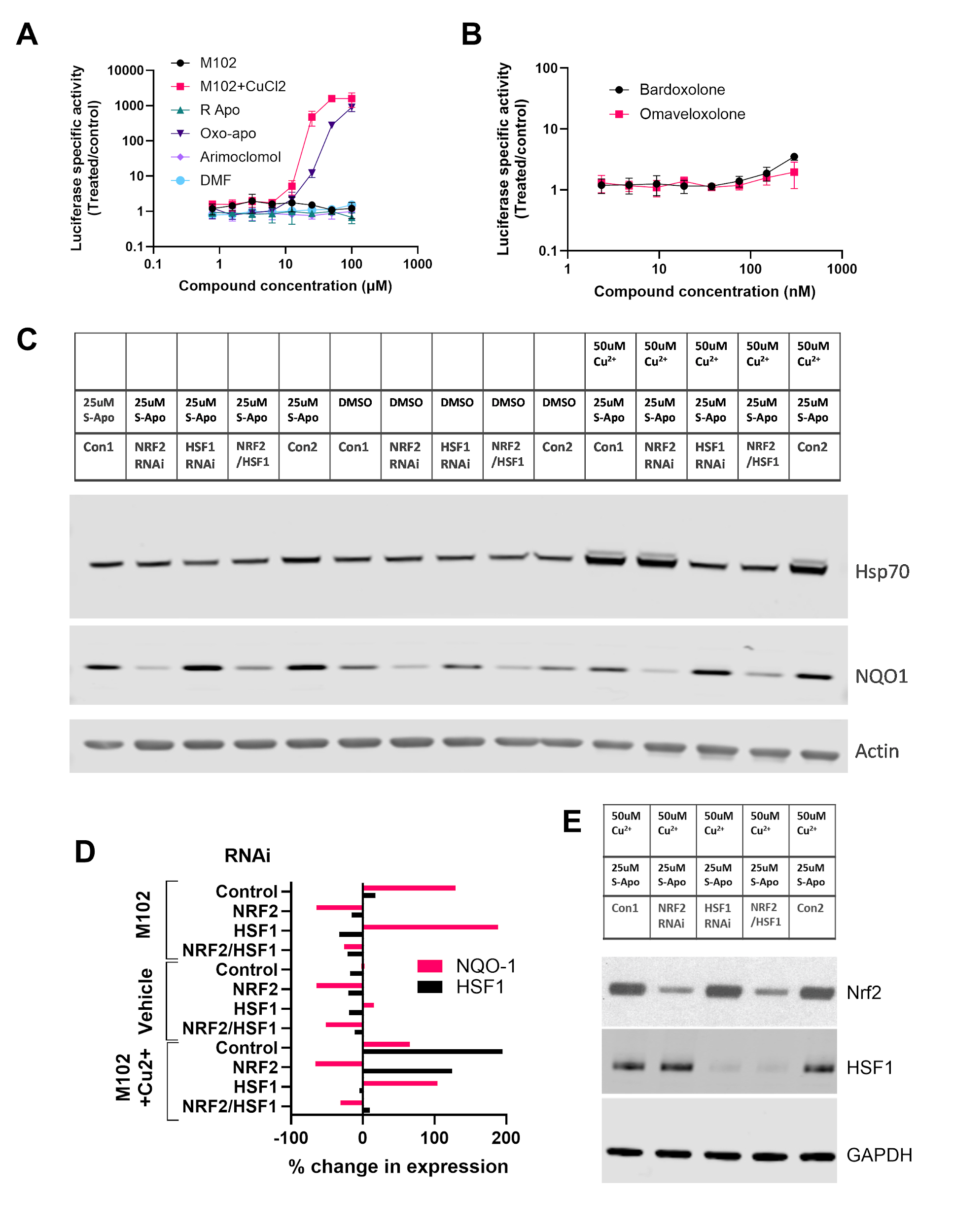


**Supplementary Figure 1: M102 activates HSF1 directed transcription of an HSPA1A (HSP70)-driven reporter.**  A, B) M102 (S-Apomorphine), R[-]apomorphine (R Apo), oxoapomorphine (Oxo-Apo), arimoclomol and Tecfidera (dimethyl fumarate-DMF) were evaluated for the capacity to induce HSF1-directed transcription in HeLa cells stably transfected with a Hsp70.1 promoter driving luciferase expression. M102 in the presence of CuCl_2_, or an oxidised from of M102 (oxoapomoprhine), show robust activation of HSF1-directed transcription. Tecfidera and arimoclomol show minimal HSF1 activation, although it is known that arimoclomol is a co-inducer of the heat shock response. B) The NRF2 activators bardoxolone and omaveloxolone do not induce the heat shock response. Note that compounds were tested in concentration ranges correlating to their known NRF2 inducing potencies. Bardoxolone and Omaveloxolone are considerably more potent electrophiles which necessitated testing them at a lower concentration range. C) Western blotting of NRF2 target NQO1 and the HSF1 target HSP70 in SHSY5Y cells following treatment with 25μM M102 or M102 plus CuCl_2_ combined with RNAi to reduce expression of HSF1 and/or NRF2. D) Analysis of relative densitometries of NQO1 or HSP70 bands over Actin from C) shows robust induction of NQO1 by M102 or M102+CuCl_2_ and robust induction of HSP70 by M102 + CuCl_2_. The induction of HSP70 is ablated by treatment of the cells with RNAi directed at HSF1 and likewise, the induction of NQO1 is ablated by treatment with RNAi directed at NRF2. E) Confirmation of HSF-1 and NRF2 by the respective RNAi treatments alone or in combination in M102 (S-Apomorphine) treated cells.

**
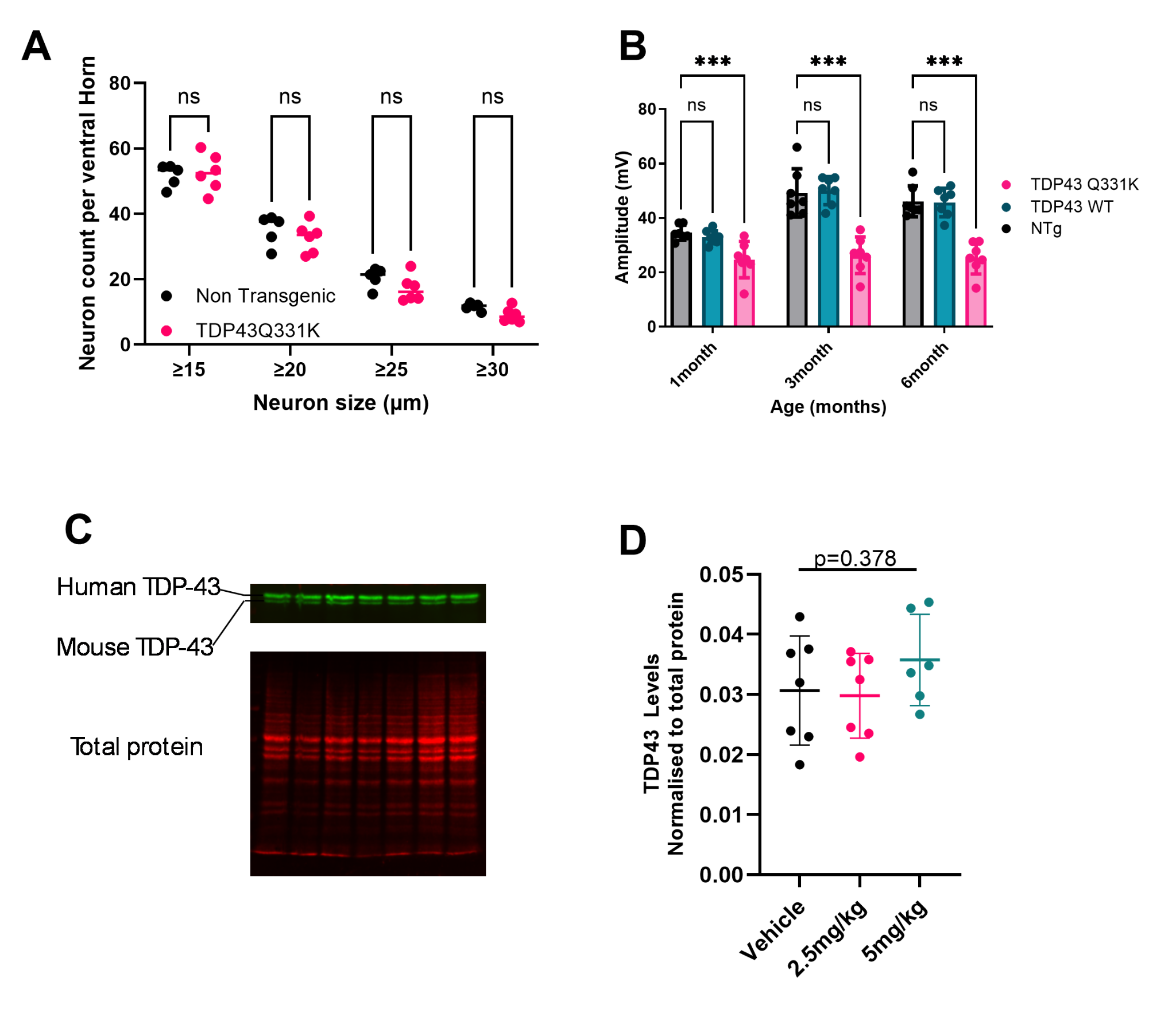
**

**Supplementary Figure 2: A)** Lumbar ventral horn motor neuron counts in TDP-43^Q331K^ transgenic and non-transgenic littermates**.** Motor neurons were counted as described in the supplementary methods in size bins ranging from >15 to >30 microns (single chord length within cytoplasm) from spinal cord tissue collected from 5 month old mice. No significant differences were seen between the *TDP-43*^Q331K^ transgenic mice and non-transgenic littermates. Two-way ANOVA overall p=0.36 for effect of genotype. B) CMAP amplitude in *TDP-43*^Q331K^ transgenic mice and littermate non-transgenic mice and *TDP-43*^WT^ transgenic mice at 1, 3 and 6 months of age. *TDP-43*^Q331K^ mice show a significant reduction in CMAP amplitudes at all time points compared to non-transgenic mice, with an ~50% reduction in CMAP at 3 and 6 months of age. (Mixed effects analysis, *p<001, N=7-8 per group). C ) Representative western blotting for TDP-43 in cortex tissue of M102 treated TDP-43^Q331K^ mice at 6 months of age and D) corresponding quantification normalised to total protein showing no significant differences between vehicle and M102 dosed groups (p=0.378, one Way ANOVA, n=6-7 per group).

**
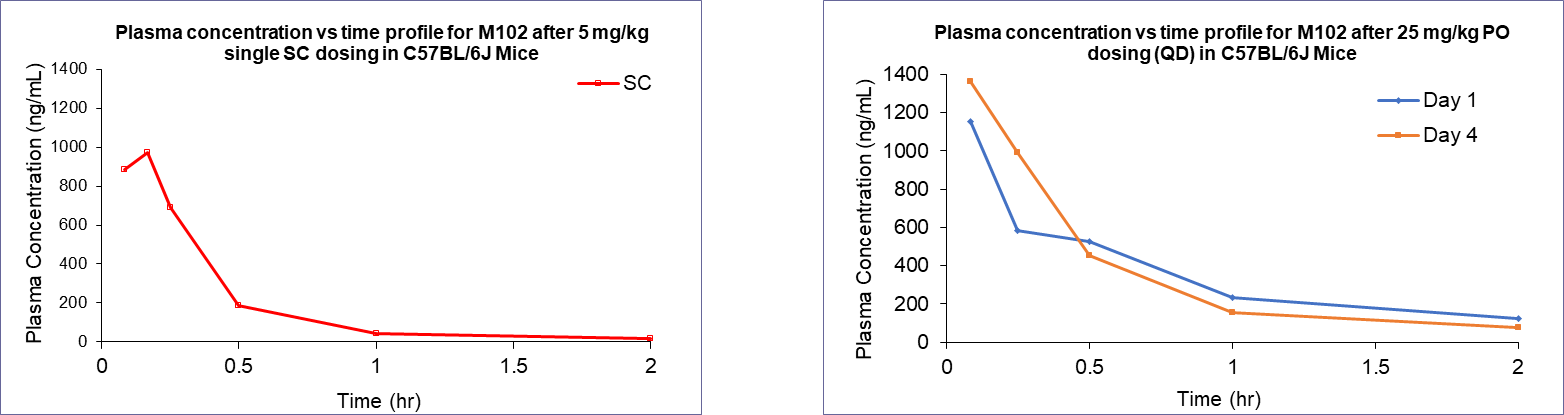
**

**
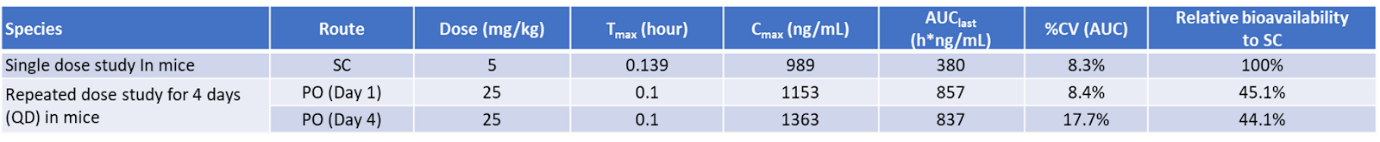
**

**Supplementary Figure 3**: **Oral gavage dose of 25mg/kg is bioequivalent to 5mg /kg subcutaneous dose in mice.** A) Pharmacokinetic study of M102 following single subcutaneous (sc) dose at 5mg /kg or B) oral dosing for 1-4 days at 25 mg/kg/day PO (QD). Data show plasma concentrations as determined by HPLC-MS , which was equivalent to 5mg/kg/day SC, assuming 20% relative bioavailability in the multiple dose study. Day 4 showed consistent PK profile to Day 1 Pharmacokinetic profile of M102 dosed at 5mg/kg SC and 10 mg/kg PO. Multiple oral dose study showed consistent exposure.

**
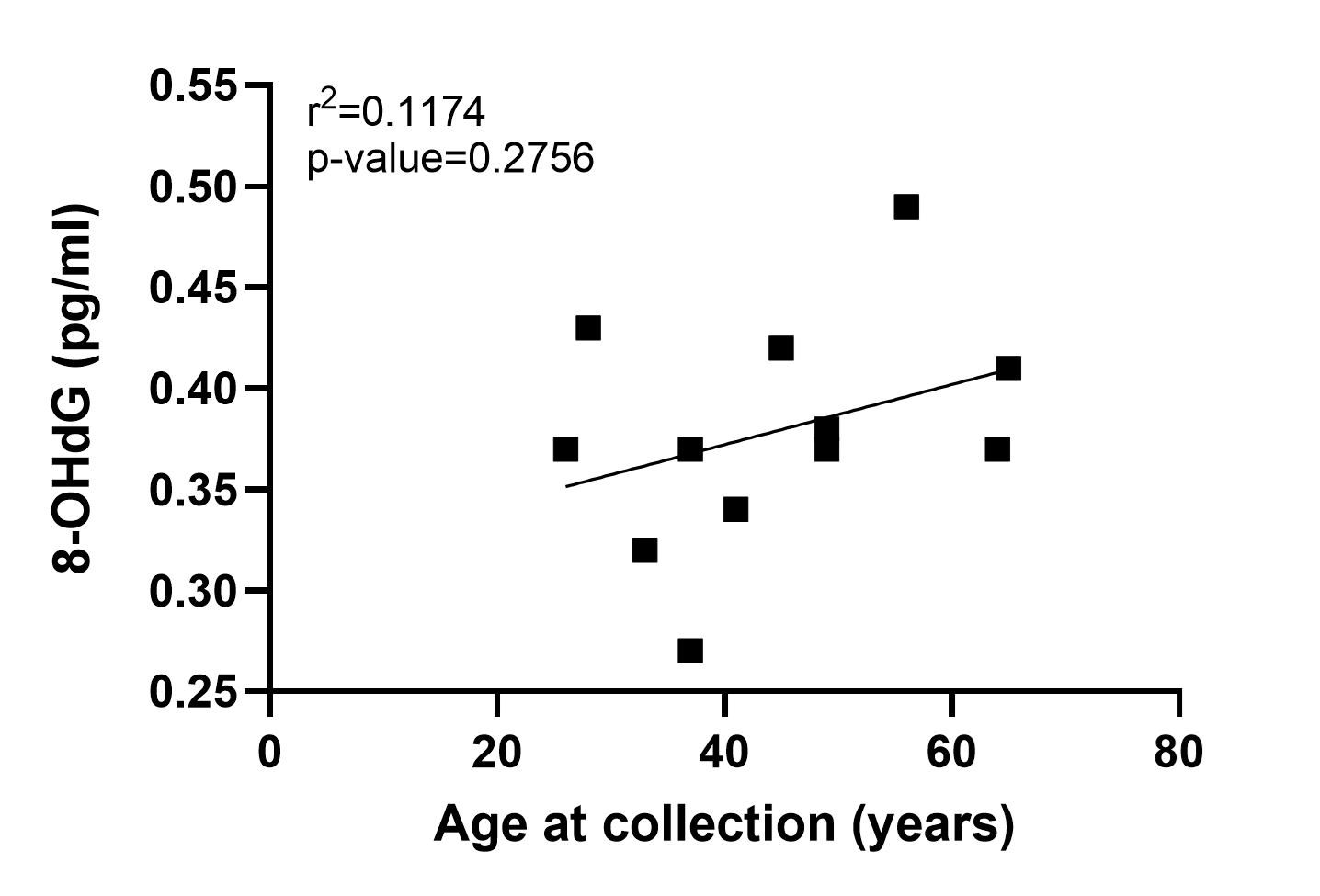
**

**Supplementary Figure 4: The levels of oxidised RNA in the CSF do not correlate with age in healthy controls.** Pearson correlation. N=11.


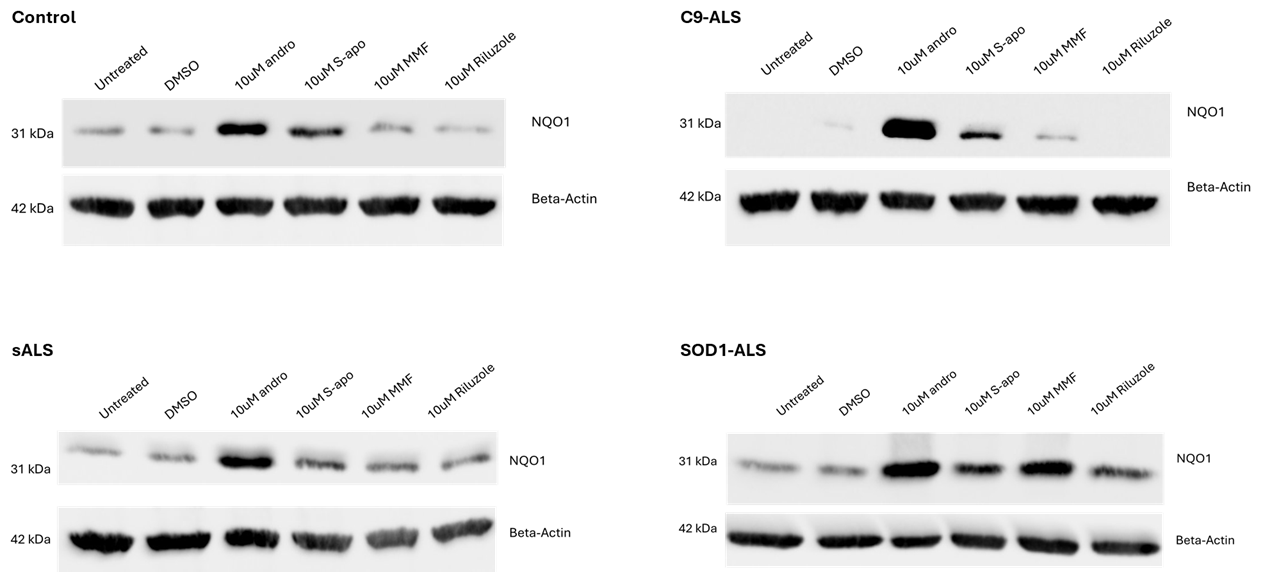


**Supplementary Figure 5:** Full western blot membranes showing NQO1 and Beta-actin expression under baseline conditions and after 48h treatment with M102 (S-apomorphine) treatment.


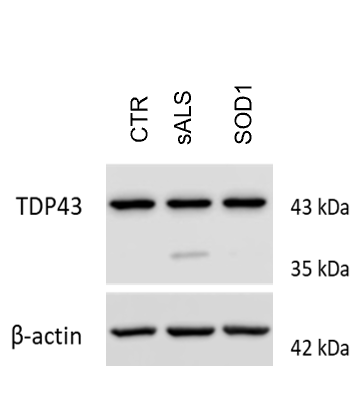


**Supplementary Figure 6:** Representative image of immunoblotting showing that iAstrocytes derived from a *SOD1*-ALS patient do not display the fragmented 35kDa TDP43 band whereas iAstrocytes derived from a sALS patient display the fragmented 35kDa band, characteristic of TDP43 proteinopathy.


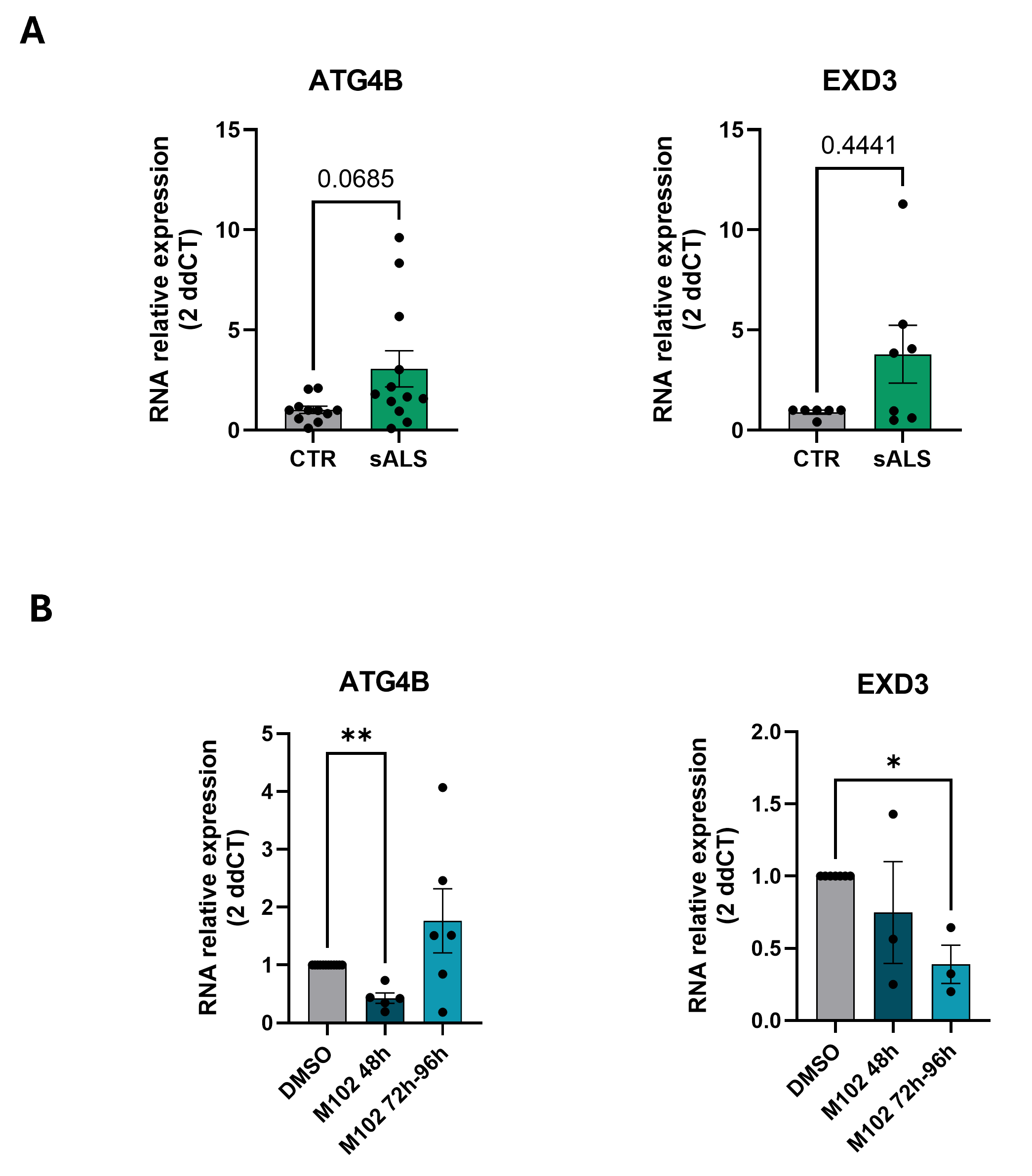


**Supplementary Figure 7: M102 treatment reverses TDP-43 regulated cryptic splicing in sALS iAstrocytes.** **A)** Cryptic splicing in the two genes with high astrocytic expression, ATG4B and EXD3, in control and sALS iAstrocytes, as analysed by RT-qPCR. Note a trend towards accumulation of cryptic exon-containing isoforms in sALS iAstrocytes compared to control lines. **B)** M102 treatment leads to reduced cryptic exon inclusion for both genes, with different temporal dynamics. Combined data for 3 control lines and 3 sALS lines are shown; data-points correspond to technical replicates. *p<0.05, **p<0.01, A) Mann-Whitney U test, B) Kruskal-Wallis test followed by multiple comparisons.

**
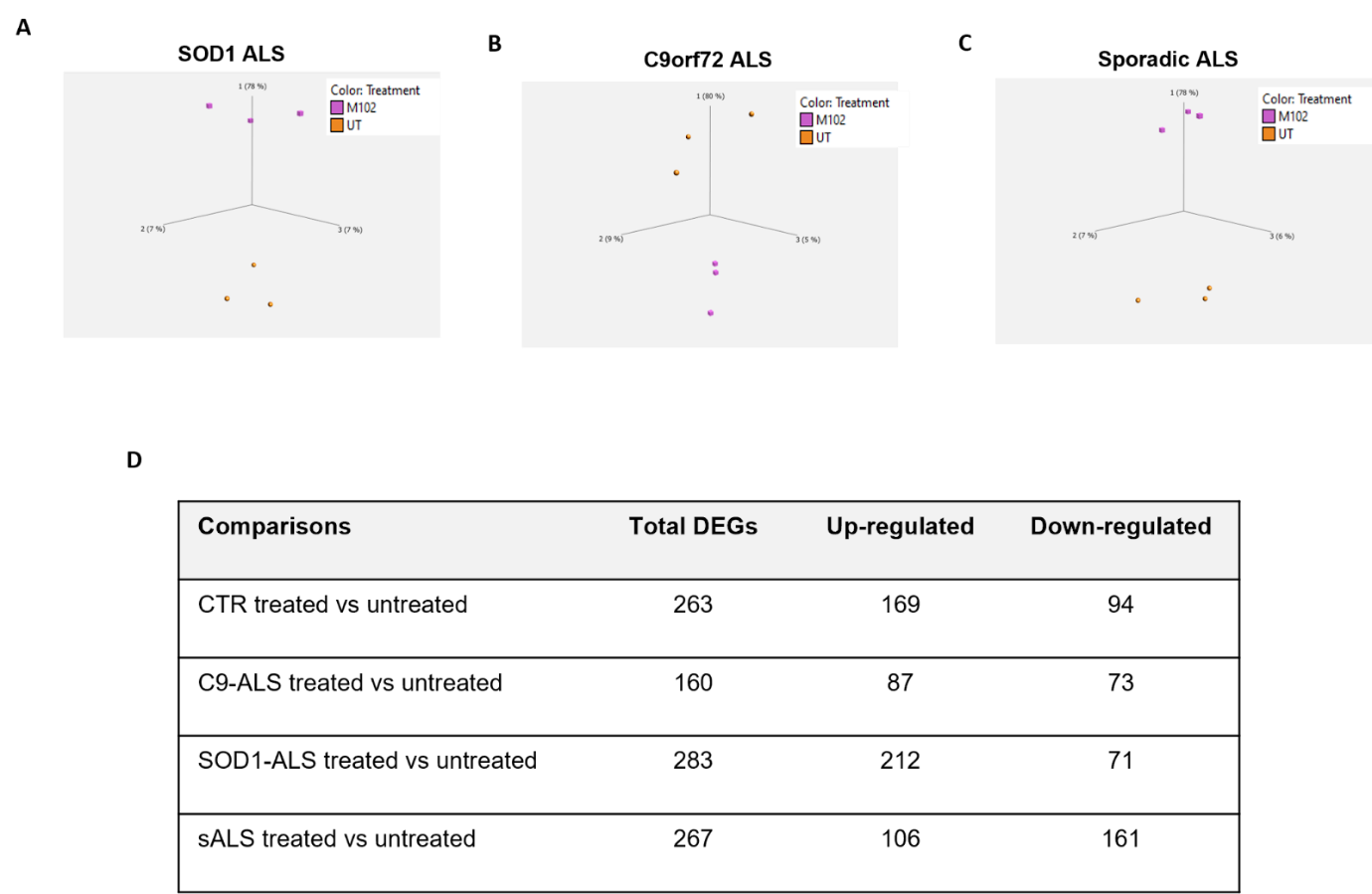
**

**Supplementary Figure 8**:  **RNA sequencing from iAstrocytes treated with 10 μM M102 or DMSO for 48h reveal genotype specific transcriptomic changes in response to M102 treatment.** These transcriptomic changes are observed across: A) *SOD1*, B) *C9orf72*, and C) sporadic ALS cases, suggesting that M102 has the potential to be beneficial for both familial and sporadic ALS patients. D) Summary of the DEGs identified between M102 treated and untreated iAstrocytes from healthy controls, *C9*-ALS, *SOD1*-ALS and sALS cases.

**Supplementary Table 1**: **Brain penetration of M102 in male Sprague-Dawley rats.**

| **Sampling time post M102 single oral dose at 12 mg/kg** | **M102 plasma concentration (ng/mL)** | **M102 brain concentration in (ng/mL)** | **Brain-to-plasma Ratio** |
| --- | --- | --- | --- |
| 30 min | 43.27 | 30.28 | 0.70 |
| 60 min | 40.12 | 14.54 | 0.36 |

**Supplementary Table 2: M102 28-day GLP rat toxicology study summary findings.**

| **Study** | **M102: 28-day oral gavage toxicity and toxicokinetics study in rats with a 14-day recovery phase** |
| --- | --- |
| **Animal** | Sprague-Dawley rats. Animals were approximately 6-7 weeks of age with body weights ranging from 178.46 to 211.76 g in females and 252.21 to 295.14 g in males at dosing initiation. |
| **Study design** | In total 142 rats (71 per sex) were randomly assigned to 4 groups, i.e., Groups 1-4 with M102 dose levels by oral gavage at 0, 25, 50, and 75 mg/kg, respectively. For the main toxicity study animals were 10/sex/group, while Groups 1 and 4 had an additional 5/sex/group to assess the recovery of any observed effects. TK animals were 3/sex in the control group and 6/sex/group in the treated groups. |
| **Evaluation criteria** | Viability (morbidity/mortality), clinical observations, body weight, food consumption, ophthalmology, clinical pathology (hematology, coagulation, serum chemistry, and urinalysis), toxicokinetics (TK), gross pathology, organ weights, and histopathology. |
| **Observation** | There were no test article-related deaths during the study. There were no test-article related body weight, food consumption, ophthalmologic, clinical pathology (hematology, coagulation, serum chemistry and urinalysis) changes and gross examination noted at any dose level. |
| **Pathology** | Test article-related anatomic pathology findings were present in the thyroid glands and kidneys, and both were considered non-adverse. Thyroid findings consisted of minimal weight reductions in males at all doses (with a weak dose response) and in females at ≥50 mg/kg/day, with no dose response. The change was reversible in females but not in males. Lower thyroid weights had no microscopic or discernible functional correlates, and therefore, were not considered adverse. Kidney findings consisted of mild tubular dilation within the renal medulla in a single female at 75 mg/kg/day. This change was considered non-adverse on the basis of the good clinical condition of this animal and the absence of clinical pathology correlates. The change was not present in any recovery animals, but reversibility could not be assessed due to the low incidence in the dosing phase and the smaller number of recovery animals available for evaluation. |
| **Toxicokinetics** | TK of M102 was evaluated on Day 1 and Day 28. Tmax values were observed at 0.5 hours postdose in animals given ≤50 mg/kg/day and females at 75 mg/kg/day on Day 1 as well, and at 0.3 hours postdose at 75 mg/kg/day (except for females on Day 1). No marked sex difference in systemic exposure was observed, except that females had higher systemic exposure than males at 25 mg/kg/day on Day 1. The systemic exposure increased dose proportionally in males on Day 1 and in both sexes on Day 28, but decreased in females on Day 1 as the dosage increased from 25 to 75 mg/kg/day. No marked drug accumulation in systemic exposure was observed, except that an increase in systemic exposure was observed at 75 mg/kg/day in males. |
| **Conclusions** | M102 when administered to rats at 25, 50 and 75 mg/kg/day by oral gavage once daily for 28 days followed by a 14-day recovery period was well tolerated and had no adverse findings in pre-/ante-mortem assessment. Test article-related anatomic findings were seen within thyroid glands at ≥50 mg/kg/day and kidneys at 75 mg/kg/day. However, these findings were considered non-adverse due to the absence of impact on animals’ general well-being or functional or clinical pathologic correlations. Therefore, under the conditions of this study, the no observed adverse effect level (NOAEL) of M102 was considered to be 75 mg/kg/day. At the NOAEL, plasma exposures, represented by maximum concentration (Cmax) and area under the concentration-time curve from time 0 through 24 hours (AUC0-24h) of M102 following the last dose, were 1010 ng/mL and 4970 ng*h/mL, respectively, in males and 668 ng/mL and 4120 ng*h/mL, respectively, in females. |

**Supplementary Table 3: M102 28-day GLP non-human primate (NHP) toxicology study.**

| **Study** | **M102: 28-day oral gavage toxicity and toxicokinetics study in NHPs with a 14-day recovery phase** |
| --- | --- |
| **Animal** | Cynomolgus monkeys. Animals were approximately 2.4 to 2.9 months of age and with body weights ranging from 2.1 to 3.8 kg in males and 2.1 to 2.9 kg in females at dosing initiation. |
| **Study design** | In total 32 (16/sex) NHPs were randomly assigned to 4 groups including 5/sex/group in control and high dose groups, and 3/sex/group in low and middle dose groups. Control or M102 in vehicles at doses of 25, 50, or 75 mg/kg/day. At the end of the dosing phase, the last 2 surviving NHPs/sex/group in control and high dose groups were held for an additional 14 days without administration of the test article. |
| **Evaluation criteria** | Viability (morbidity/mortality), clinical observations, body weight, food consumption, ophthalmic examinations, electrocardiograms, clinical pathology (hematology, serum chemistry, coagulation, urinalyses), gross (necropsy) evaluation, organ weight, histopathological evaluation and toxicokinetics. |
| **Observation** | No test article-related changes in clinical signs, body weight/body weight gain, food consumption, ophthalmologic examinations, electrocardiograms, clinical pathology (hematology, serum chemistry, coagulation, and urinalysis), organ weights, gross lesions and microscopic pathology at any dose level during the dosing and recovery phases. |
| **Toxicokinetics** | After once daily oral administration of M102 at 25, 50, or 75 mg/kg/day to male and female monkeys for 28 days, median Tmax values were observed at 1.0 and 2.0 hours postdose. Median T1/2 values ranged from 3.1 and 6.2 hours. No marked sex difference in systemic exposure was observed except that females had higher systemic exposure than males at 50 mg/kg/day on Day 28. The systemic exposure increased dose-proportionally in both sexes on Days 1 and 28 as the dosage increased from 25 to 75 mg/kg/day. No marked drug accumulation was observed at any dose level. |
| **Conclusion** | Administration of M102 to monkeys once daily for up to 28 days by oral gavage at dosages of 25, 50 or 75 mg/kg/day followed by a 14-day recovery period was well tolerated and did not result in any treatment-related mortality, or any test article-related effects in clinical signs, body weight, food consumption, ophthalmology, electrocardiography, clinical pathology and pathology. The no observed adverse effect level (NOAEL) for this study was considered to be 75 mg/kg/day. The corresponding AUC0-24h and Cmax of M102 following the last dose at NOAEL were 1350 h*ng/mL and 319 ng/mL for males, and 901 h*ng/mL and 209 ng/mL for females, respectively. |

**Supplementary Table 4:** **Post-mortem CNS tissue used for 8-OHG staining**

| **Case ID** | **Gender** | **Age** | **PMD** | **Diagnosis** | **Comments** |
| --- | --- | --- | --- | --- | --- |
| 98-07 | M | 67 | 63hrs | Hepatocellular carcinoma |  |
| 10-96 | F | 63 | 31hrs | Carcinoid Tumor |  |
| 07-70 | M | 26 | Not available | Epilepsy (sudden death) | No structural pathologic abnormality |
| 05-64 | M | 66 | 96hrs | sALS |  |
| 08-24 | F | 63 | 36hrs | sALS |  |
| 08-96 | F | 69 | 40 hrs | sALS |  |
| 14-11 | M | 51 | 40hrs | sALS |  |
| 05-42 | M | 66 | 9hrs | sALS |  |
| 59-09 | F | 80 | N/A | sALS |  |
| 69-09 | M | 48 | 23hrs | fALS C9orf72 |  |
| 83-10 | M | 79 | 13hrs | fALS C9orf72 |  |
| 41-04 | M | 79 | 13hrs | ALS+FTD C9orf72 |  |
| 66-08 | F | 59 | 28hrs | fALS+FTD C9orf72 |  |

**Supplementary Table 5**: **CSF samples used for the 8-OHG ELISA**

| **Sample** | **Disease** | **Gender** | **Date of birth** | **Date of collection** | **Age at collection** |
| --- | --- | --- | --- | --- | --- |
| 3624 | Sporadic ALS | Male | 07/02/1962 | 20/11/2015 | 53 |
| 3629 | Sporadic ALS | Male | 29/04/1946 | 10/12/2015 | 69 |
| 427 | Sporadic ALS | Male | 05/09/1950 | 13/02/2014 | 63 |
| 3392 | Sporadic ALS | Female | 11/02/1951 | 20/11/2014 | 63 |
| 240 | Sporadic ALS | Male | 25/01/1952 | 21/03/2006 | 54 |
| 248 | Sporadic ALS | Male | 29/04/1940 | 27/03/2006 | 65 |
| 254 | Familial ALS (mutation unknown) | Male | 03/10/1961 | 11/04/2006 | 44 |
| 303 | Sporadic ALS, MS | Female | 28/01/1943 | 10/10/2006 | 63 |
| 3575 | Sporadic ALS | Female | 26/11/1967 | 27/08/2015 | 47 |
| 3543 | Sporadic ALS | Male | 08/04/1953 | 06/07/2015 | 62 |
| 2307 | Familial ALS (M337V TARDBP mutation) | Female | 15/06/1973 | 09/07/2015 | 42 |
| 3624 | Sporadic ALS | Male | 07/02/1962 | 20/11/2015 | 53 |
| 3653 | Sporadic ALS | Male | 14/05/1975 | 17/02/2016 | 40 |
| C3631 | Healthy control | Male | 30/10/1959 | 15/12/2015 | 56 |
| C3634 | Healthy control | Female | 21/06/1950 | 01/02/2016 | 65 |
| C209 | Control:Headache | Female | 03/08/1955 | 06/06/2005 | 49 |
| C347 | Control:Depression | Male | 21/09/1970 | 09/02/2008 | 37 |
| C245 | Control:Facial weakness | Female | 03/07/1950 | 27/11/2014 | 64 |
| C3619 | Control:Headache | Female | 28/08/1987 | 17/11/2015 | 28 |
| C3606 | Control:Migraine | Female | 07/02/1970 | 14/10/2015 | 45 |
| C3549 | Control:Migraine | Female | 11/05/1978 | 22/07/2015 | 37 |
| C3548 | Healthy control | Female | 08/06/1989 | 15/07/2015 | 26 |
| C3576 | Healthy control | Male | 28/10/1973 | 01/09/2015 | 41 |
| C387 | Control: Headache | Male | 28/03/1975 | 21/10/2008 | 33 |
| C355 | Control: Functional gait disorder | Male | 16/10/1959 | 09/09/2008 | 49 |

**Supplementary Table 6:** **Cell lines used for *in vitro* experiments**

| **Cell line** | **Genetic remarks** | **Sex** | **Age at biopsy collection (years)** | **Ethnicity** | **Onset to death (months)** |
| --- | --- | --- | --- | --- | --- |
| CTR_1 | Non-ALS control | Male | 40 | Caucasian | - |
| CTR_2 | Non-ALS Control | Male | 31 | Caucasian | - |
| CTR_3 | Non-ALS control | Female | 64 | Caucasian | - |
| CTR_4 | Non-ALS control | Male | 68 | Caucasian | - |
| CTR_5 | Non-ALS control | Female | 69 | Caucasian | - |
| sALS_1 | sALS | Female | 61 | Caucasian | 21 |
| sALS_2 | sALS | Male | 29 | Caucasian | 90 |
| sALS_3 | sALS | Male | 47 | Caucasian | 72 |
| sALS_4 | sALS | Male | 59 | Caucasian | - |
| sALS_5 | sALS | Female | 85 | Caucasian | 25 |
| sALS_6 | sALS | Female | 80 | Caucasian | 29 |
| sALS_7 | sALS | Male | 54 | Caucasian | 37 |
| sALS_8 | sALS | Female | 58 | Caucasian | - |
| sALS_9 | sALS | Male | 56 | Caucasian | 49 |
| sALS_10 | sALS | Male | 65 | Caucasian | - |
| C9_1 | C9orf72 ALS | Male | 66 | Caucasian | 31.7 |
| C9_1 | C9orf72 ALS | Male | 50 | Caucasian | 27 |
| C9_3 | C9orf72 ALS | Female | 66 | Caucasian | 19.4 |
| SOD1_1 | SOD1 (A4V) | Female | 63 | Caucasian | - |
| SOD1_2 | SOD1 (A4V) | Female | 40 | Caucasian | - |
| SOD1_3 | SOD1 (D90A) | Male | 56 | Caucasian | - |
| SOD1_4 | SOD1 (D76Y) | Female | 42 | Caucasian | 81 |

**Supplementary Table 7**: **List of GO pathways shared between ALS patient subgroups treated with M102.**

| Gene Ontology Term | CTR p-value | C9ORF72 p-value | SOD1 p-value | sALS p-value |
| --- | --- | --- | --- | --- |
| Cellular response to organic cyclic compound | 4.10E-03 | - | 2.20E-04 | - |
| MAPK cascade | 2.70E-02 | - | 3.60E-02 | - |
| Morphogenesis of an epithelial fold | 4.40E-02 | - | - | 4.00E-02 |
| Cell adhesion | - | 3.40E-05 | 2.80E-02 | 4.30E-02 |
| VEGF pathway | - | 3.20E-02 | 1.80E-02 | 2.50E-02 |
| Inflammatory response | - | 6.20E-04 | 5.40E-05 | 3.80E-02 |
| Autophagic response | - | 2.40E-04 | 1.90E-03 | 2.70E-02 |
| Oxidation-reduction process | - | 5.60E-03 | 5.00E-02 | 3.70E-02 |
| Xenobiotic metabolic process | 5.60E-02 | - | 7.90E-04 | 4.40E-02 |
| Synaptic transmission, cholinergic | - | - | 3.70E-04 | 6.10E-03 |
| Collagen fibril organisation | - | - | 5.60E-03 | 6.20E-04 |

**Supplementary Table 8**: **List of high and low responders to M102**

|  | **Cell line** | **p-value DMSO vs M102** | **% improvement in MN survival in co-culture** |
| --- | --- | --- | --- |
| **High responders** | C9-ALS 183  SOD1 ALS 210  SOD1 ALS 102  *SOD1 ALS 100  sALS 009 | <0.0001  <0.0001  <0.0001  0.0008  <0.0001 | 85.9%  97.1%  83.3%  61.1%  76.1% |
| **Low responders** | C9-ALS 78  C9-ALS 201  sALS 12  sALS 17 | 0.0113  0.0748  0.8931  0.0199 | 33.9%  31.7%  27%  19.1% |

**Supplementary Table 9** **:** **Primary antibody for immunohistochemistry of FFPE**

| **Antibody** | **Species** | **Dilution factor and conditions** | **Antigen retrieval** | **Supplier** |
| --- | --- | --- | --- | --- |
| 8-OHdG | Mouse monoclonal | 1/400 1h RT | Pressure cooker access revelation (×10, pH 6.5) | Abcam, Cambridge UK  #ab10802 |

**Supplementary Table 10** : **Primary and secondary antibodies for immunocytochemistry of iAstrocytes**

| **Primary** | **Species** | **Dilution factor** | **Supplier** |
| --- | --- | --- | --- |
| 8-OHdG | Mouse monoclonal | 1:100 | Abcam; #ab10802 |
| NRF2 | Rabbit polyclonal | 1:500 | Abcam; #ab31163 |
| NRF2 | Rabbit polyclonal | 1:200 | Biorbyt; #ORB128433 |
| HSF1 | Rabbit polyclonal | 1:500 | Cell Signaling Technologies; #4356 |
| mSOD1 | Mouse monoclonal | 1:100 | Medimabs; #4356 |
| **Secondary** | **Species** | **Concentration** | **Supplier** |
| anti-chicken Alexa 488 | Goat | 2mg/ml | Invitrogen; A11039 |
| anti-rabbit Alexa 488 | Goat | 2mg/ml | Invitrogen; A11008 |
| anti-rabbit Alexa 568 | Donkey | 2mg/ml | Invitrogen; A10042 |
| anti-mouse Alexa 568 | Donkey | 2mg/ml | Invitrogen; A10037 |

**Supplementary Table 11:** **Primary antibodies for immunoblotting of iAstrocytes**

| **Antibody** | **Species** | **Dilution and conditions** | **Supplier** |
| --- | --- | --- | --- |
| TDP-43 | Rabbit polyclonal | 1:1000 | Proteintech; 12892-1-AP |
| NQO1 | Rabbit polyclonal | 1:1000 | Abcam; #ab34173 |
| B-actin | Mouse  monoclonal | 1:10,000 | Abcam; #ab6276 |
